# Porous Thermoformed Protein Bioblends as Degradable Absorbent Alternatives in Sanitary Materials

**DOI:** 10.1021/acsapm.3c01027

**Published:** 2023-08-25

**Authors:** Agnès Jugé, Jeannine Moreno-Villafranca, Victor M. Perez-Puyana, Mercedes Jiménez-Rosado, Marcos Sabino, Antonio J. Capezza

**Affiliations:** † KTH Royal Institute of Technology, Department of Fibre and Polymer Technology, Polymeric Materials Division, School of Engineering Sciences in Chemistry, Biotechnology and Health, Stockholm 10044, Sweden; ‡ B5IDA Research Group Chemistry Department, Universidad Simón Bolívar, AP 89000, Caracas, Venezuela; § University of Seville, Department of Chemical Engineering, Seville 41012, Spain

**Keywords:** bioblends, porous materials, protein absorbents, sanitary materials, sustainable materials, biodegradability

## Abstract

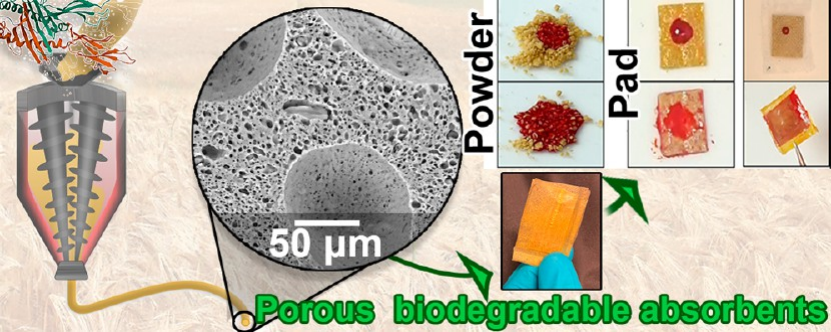

Protein-based porous absorbent structures can be processed and assembled into configurations suitable for single-use, biodegradable sanitary materials. In this work, a formulation based on a mixture of proteins available as industrial coproducts is processed into continuous porous structures using extrusion and assembled using conventional thermal methods. The experimental design led to formulations solely based on zein-gluten protein bioblends that could be manufactured as liquid absorbent pellets, compressed pads, and/or porous films. The processing versatility is attributed to the synergistic effect of zein as a low viscosity thermoformable protein with gluten as a readily cross-linkable high molecular weight protein. The capillary-driven sorption, the biodegradability of the materials, and the possibility to assemble the products as multilayer components provide excellent performance indicators for their use as microplastic-free absorbents. This work shows the potential of biopolymers for manufacturing sustainable alternatives to current nonbiodegradable and highly polluting disposable items such as pads and diapers.

## 1 Introduction

From early childhood, we use massive amounts of disposable absorbent sanitary products such as diapers, napkins, and incontinence products.^1,2^ Studies have shown that only one newborn baby can produce up to 1 ton of waste in diapers, while estimations show that an elderly care home with 20 residents produces *ca.* 90 L of waste daily only on incontinence diapers.^3^ Although estimations from the total environmental impact of disposable sanitary products vary, only in the United Kingdom, the largest environmental pollutant among disposable products is sanitary pads contributing to 6.600 tCO_2_ equiv of greenhouse gas (GHG) emissions.^4^ The market for sanitary products increases with the world population and the convenience of disposable materials in our daily life. Despite the ban on single-use fossil-based plastics and the known effects of the plastics used to produce superabsorbent polymer gels (SAP), these products are still built on these materials.^5^ Recent studies detected toxic carcinogenic, mutagenic, endocrine disrupting substances, and even microplastics lixiviating from disposable sanitary items exposed in nature.^6^ In addition, the life cycle of these products is completely nonsustainable, from the choice of raw materials from petroleum to their end-of-life reliance on plastics degrading into microplastics.^6−10^ New technologies ensuring the use of biomass from current industrial processes, efficient environmentally friendly transformation processes, and biodegradable materials safe for humans and nature are of utmost importance to ensure the sustainable production of future sanitary articles with minimal environmental impact.

Using industrial biomass as raw material to produce bioplastics with sorbent properties is the current most promising experimental route to avoid exploiting virgin recourses such as cotton (also used in sanitary pads).^11^ Extensive literature is available in superabsorbent hydrogels from biomass such as cellulose and nanocellulose-based composites.^12−16^ Recent studies include the use of proteins as coproducts from the starch industry to produce biomaterials with conventional polymer processing techniques (extrusion, injection, compression molding) with high degradation rates and bioassimilation.^17−20^ The potential of using protein-based formulations with high bioassimilation ensures that the product maintains a safe end-of-life scenario even if accidentally disposed of in nature.^1^ Nonetheless, an important drawback is that the reported protein formulations resulted in higher CO_2_ emissions than conventional absorbents because of the use of chemicals to increase the liquid absorption properties of the proteins.^1^ The reports also show that the processing led to nonhomogenous porous absorbent structures and only focused on the absorbent material.^20^ Considering that a sanitary item is assembled by combining an absorbent core and nonwoven polyethylene/polypropylene fibers, attention shall be put to integrated solutions where both elements are combined toward a fully sustainable system.

This work reports the production of stable porous protein absorbent layers based on chemical-free bioblends processed at low temperatures and displaying high processability in multiple polymer processing techniques. The remarkable processability resulted from the combination of gluten and zein protein as a functional bioblend and is demonstrated by the ability to produce continuous porous extrudates, pressed pellets, and porous film structures from the same formulation. The different layers built are readily assembled into a prototype having functional free liquid absorption, spreading, and retention properties resembling a reference material based on PE/PP nonwoven films encapsulating a porous polyurethane foam. The proposed formulation allows preparing porous liquid absorbent networks that are fully biodegradable in less than one month, do not require synthetic layers or cotton (as current sanitary disposable items), and are rapidly degradable into safe molecules demonstrated using commercial reference products. The concept allows for the design of all-in-one disposable items (absorbents encapsulated in permeable external layers), and the simplicity of the manufacturing strategy increases the potential for pilot-scaling in several products, such as sanitary pads, medical patches, etc.

## 2 Materials and Methods

Concentrated wheat gluten powder (WG) was provided by Lantmännen Reppe (Sweden). The reported protein content is 86%, following the NMKL 6:2003 standard (USA) and using an N factor of 6.25. Zein protein from maize (Z) was purchased from Sigma-Aldrich (Sweden). The reported protein content is 88–96%. Glycerol (99%) and sodium bicarbonate (NaHCO_3_, >99.7%) were purchased from Fisher Scientific (Sweden) and Sigma-Aldrich (Sweden), respectively.

### 2.1 Production of Porous Protein-Based Absorbent Structures

#### 2.1.1 Experimental Design

Preliminary mixtures of zein:gluten (Z:WG) with different glycerol amounts were prepared to decide on representative protein and glycerol ratios, allowing the production of malleable mixtures with good consistency (Table S1). The consistency and appearance of the mixtures were evaluated using a WG with a 50% glycerol recipe, which has been previously used for the extrusion of gluten materials.^20^ The formulations resulting in sticky or sandy mixtures were not considered. The most optimal ranges were used in the experimental design to determine key material and processing parameters to produce highly porous and continuous extrudates. The mixtures were manually premixed and then extruded in a microcompounder corotating double-screw DSM Xplore 5 (Xplore instruments, The Netherlands). The initial formulations were extruded using a circular die with a 4 mm diameter.

The experimental design was followed by using a Hadamard matrix to allow for a first evaluation of selected factors and their impact on the material properties (response). The factors set were (i) WG:Z ratio, (ii) glycerol content, (iii) water content, (iv) extrusion temperature, (v) extrusion speed, and (vi) sodium bicarbonate amount. Once the factor was selected, the level was decided to be −1 or +1, according to Table 1.

**Table 1:** Design of the Experimental Factors Used to Construct the Hadamard Matrix

		level	
number	factor	+1	–1
X1	WG:Z ratio	75:25	25:75
X2	Glycerol content (wt %)^a^	60	30
X3	Water content (wt %)^a^	5	0
X4	Extrusion temperature (°C)	100	80
X5	Extrusion speed (rpm)	60	30
X6	Sodium bicarbonate amount	5%	0%

a The content in % refers to the amount of reagent relative to the protein content.

The Hadamard matrix with *k* ≤ 7 was constructed according to the experimental factors displayed in Table 1 by using the Plackett-Burman method. The summary of the matrix with each independent column is shown in Table 2. *Y* corresponds to the result obtained after extruding each mixture (response factor). The effect of each experiment was studied by observing the microstructure of the extrudates (cross-section). The microstructure was rated from 0 to 10 according to the material’s porosity. The scoring criteria used in this study are summarized in Table S2. The porosity was chosen as a critical parameter as we relied on producing continuous and homogeneous porous structures to maximize the liquid uptake of the extrudates. The density of the materials was also chosen as an indirect and more statistical measure of the porosity of the materials.

**Table 2:** Hadamard Matrix Used for the Experimental Study^a^

sample	X1	X2	X3	X4	X5	X6	*Y*
**1**	+1 (75:25)	+1 (60)	+1 (5)	–1 (80)	+1 (60)	–1 (0%)	Y1
**2**	+1 (75:25)	+1 (60)	–1 (0)	+1 (100)	–1 (30)	–1 (0%)	Y2
**3**	+1 (75:25)	–1 (30)	+1 (5)	–1 (80)	–1 (30)	+1 (5%)	Y3
**4**	–1 (25:75)	+1 (60)	–1 (0)	–1 (80)	+1 (60)	+1 (5%)	Y4
**5**	+1 (75:25)	–1 (30)	–1 (0)	+1 (100)	+1 (60)	+1 (5%)	Y5
**6**	–1 (25:75)	–1 (30)	+1 (5)	+1 (100)	+1 (60)	–1 (0%)	Y6
**7**	–1 (25:75)	+1 (60)	+1 (5)	+1 (100)	–1 (30)	+1 (5%)	Y7
**8**	–1 (25:75)	–1 (30)	–1 (0)	–1 (80)	–1 (30)	–1 (0%)	Y8

a The values in parentheses indicate each independent level’s factors, according to Table 1. The parameters are X1 (WG:Z ratio), X2 (glycerol content in %), X3 (water content in %), X4 (extrusion temperature in °C), X5 (extrusion speed in rpm), X6 (sodium bicarbonate content in %).

The model’s coefficient and response factor (Y) was determined by the algebraic sum of the responses (using eq 1) and dividing by the number of experiments.
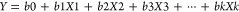
1where *Y* is the response factor (numerical), *Xk* is the respective parameter, and *bk* is the coefficient (level) of the parameter. By classifying the *bk* in decreasing order, we can evaluate the parameters with the most weight toward the final result (increasing the material’s porosity).

The Hadamard matrix allows the response *Y* to become a random variable, similar to a regression coefficient characterized by an average and standard deviation of the average. Thus, the coefficient follows a student’s *t*-distribution characterized by a mean, a standard deviation n.d.(*bi*) and a degree of freedom. The confidence interval for ″*bi*″ is calculated from eqs 2 and 3.
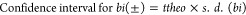
2
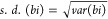
3

The material formulation derived from the experimental design was processed as porous extrudates, porous pressed pads, and porous extruded films. The materials are labeled as *x*Z/*y*WG, where Z is Zein, WG is wheat gluten, and *x*/*y* corresponds to the protein ratio in the mixture. Gly, SB, and MQ are glycerol, sodium bicarbonate, and Milli-Q water, respectively. The number before these additives corresponds to the added content and the total protein content. For instance, 75Z/25WG/40Gly/5SB/5MQ is a mixture of zein:gluten (75:25 ratio), with 40 wt % glycerol, 5 wt % sodium bicarbonate, and 5 wt % Milli-Q water, with respective to the protein content. The pilot-scale extrusion of the final recipes was performed on a single screw extruder Do-Corder C3 (Brabender, Germany).

#### 2.1.2 Porous Pressed Pads

The pads were produced by cutting the porous extruded filaments into *ca.* 0.5 cm pellets and compressing the pellets. The pellets were put in a preheated aluminum mold and made in two shapes, one rectangular (15 × 5 × 0.1) cm^3^ and one following the shape of a commercial sanitary pad (Figure S1). The mold was placed between anti-adhesion Teflon paper and preheated top and bottom plates (Figure S1). The optimal amount of material to produce a homogeneous porous shape resulted from several trials and was 0.9 g/cm^3^. The pellets were pressed at 150 °C and 150 kN in a hot-press TP-400 (Fontijne, The Netherlands). The compression cycle was 5 min under pressure + 5 min without pressure (always at 150 °C). The pressure was released after 5 min to allow the material to degas and create more porosities and, after that, cooled inside the hot-press. The pressed sample was removed from the hot-press when the temperature was below 90 °C. The molded pad was stored in a desiccator before the testing.

#### 2.1.3 Porous Films

The extruded porous films were produced in the same microcompounder used for the porous filaments but using a flat sheet die of 0.2 mm. The extrusion speed and temperature were 60 rpm and 100 °C, according to the results from the experimental design. The porosity was obtained from sodium bicarbonate (SB) or water (MQ). Solid films were also produced under the same conditions described above but without SB and MQ.

The different layers for the final proof-of-concept absorbent item prototype were assembled by using an impulse sealer for PP/PE bags (PFS-400). An absorbent reference prototype was also built by sealing a polyurethane foam with PE/PP nonwoven films from commercial menstruation pads.

### 2.3 Material Characterization

The density of the extruded materials was calculated by the gravimetric buoyancy method (Archimedes method) and an apparent density (gravimetric method, assuming a cylindrical shape). For the Archimedes method, heptane was used (ρ = 0.6838 g/cm^3^), and the densities are reported as average of triplicates.

The material microstructure from the experimental design was evaluated using a tabletop scanning electron microscope (SEM), TM-1000 (Hitachi, Japan). The samples were immersed in liquid nitrogen for 5 min and cryo-fractured. The cross-section was placed on conductive carbon tape and sputtered with Pt/Pd for 1 min before microstructural observation. The particle size distribution was determined by measuring the pore size of at least 50 pores using the software ImageJ. The microstructure of the materials produced for preparing the functional absorbent items and assembly into a sanitary article prototype was evaluated on a field-emission FE-SEM S4800 instrument (Hitachi, Japan). The samples were prepared identically as described above. The degraded material’s microstructure was compared at the initial and final degradation times using a scanning electron microscope, JSM6390 (JEOL, USA).

The liquid swelling properties of the extruded materials were determined by free swelling capacity (FSC) following the nonwoven standard procedure (NWSP) 240.0.R2 (tea bag test). A piece of the extruded material was placed in a PE/PP nonwoven plastic bag and individually immersed in a beaker with an excess saline solution (0.9 wt % NaCl). The materials were subsequently immersed in the liquid for 1, 5, 10, and 30 min. After each immersion, they were kept out of the solution for 15 s, gently placed on a paper towel for 10 s to remove the excess solution, and finally weighed. The results are reported as an average of triplicate for each immersion time. The same protocol was repeated for empty bags to calculate the correction factor (*CF*), and the FSC was estimated using eq 4.
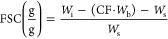
4where *W*_i_ is the weight of the bag + sample after immersion, *W*_b_ is the weight of the dry, empty bag, and *W*_s_ is the weight of the dry extruded sample.

The centrifuge retention capacity (CRC) was determined to evaluate the liquid retention within the material structure. The CRC was the ratio between FSC (30 min, saline swelling) and the weight of the material after centrifugation at 1200 rpm for 3 min (according to the NWSP 240.0.R2 standard).

The capacity of the material to absorb liquid under a constant load was determined by the absorption under load (AUL) test following the NWSP 242.0.R2 standard. The sample was placed in a cylinder having a metal grid at the bottom and closed with another containing a standard weight of 0.5 kg. The diameter of the piston pressing the sample between the metal grid and the piston is 6 cm, leaving a pressure of *ca.* 1.76 kPa (0.25 psi, equivalent to that of a newborn baby). The setup was placed on top of a porous circular ceramic plate (#0), filter paper, and then in a glass Petri dish. 180 mL of saline solution was added to the Petri dish, which is enough liquid to reach the top of the ceramic plate and allow the liquid to contact the sample via the metal grid. The sample was left under load for 1 h, removed from the Petri dish, and weighed. The experimental setup is shown in Figure S2.

The liquid spreading was determined via a visual absorption test (VAT), as reported by Capezza et al.^19^ The VAT allows observing how the fluid enters and spreads around the material and the saturation point. Aliquots of 100 μL of saline solution and defibrinated sheep blood were added to the material until saturation was reached. The VAT was the amount of liquid contained in the material divided by the dry weight of the material (g/g). The hydrophilicity and wettability of the materials were assessed using contact angle equipment Theta Lite (Biolin Scientific, Sweden). Briefly, 4 μL of MQw droplets was deposited onto the surfaces from a 200 μL tip using a sessile drop method.

The thermal properties of the zein/gluten-based bioblends were studied to evaluate the processing window of the materials and the influence of sodium bicarbonate’s addition on the protein blend’s molecular structure. In this sense, the blends’ thermal and rheological properties were analyzed on the mixtures (manually mixed) without any processing.

Thermogravimetric analyses (TGA) were performed in a Q600 calorimeter (TA Instruments, USA) between 25 and 500 °C in a nitrogen atmosphere. These tests used weight variation to evaluate the thermomechanical stability of the samples. The heating rate was set at 10 °C/min. Differential scanning calorimetry (DSC) experiments were carried out in a Q20 calorimeter (TA Instruments, USA) using hermetic aluminum pans under a nitrogen atmosphere. A 10 °C/min heating rate was used from 20 to 150 °C to evaluate the thermal transitions of each sample.

The viscoelastic properties of the materials and thermal transitions (rheological properties)^21^ were assessed via dynamic thermomechanical compression tests using a DMA805 (TA Instruments, USA) with a cylindrical plate–plate compression geometry (ϕ: 15 mm). Temperature ramps were performed from 25 to 150 °C at a heating rate of 10 °C/min. Furthermore, strain sweep tests (at 1.0 Hz, from 0.002% to 2%) and frequency sweep tests (at constant strain within the linear viscoelastic range, from 0.2 to 20 Hz) were carried out at different temperatures (20, 80, 100, and 140 °C). In these tests, the values of the viscoelastic moduli were collected (*E*′ and *E*″ corresponding to the elastic and viscous moduli, respectively), and the relationship between them (tan δ = *E*″/*E*′) and the critical strain (last strain in the linear viscoelastic range) of the systems were measured. All measurements were carried out in triplicate.

### 2.4 End-of-Life Evaluation: Hydrolytic Degradation

#### 2.4.1 Buffer Preparation

Buffers were prepared at pH 3.89, pH 6.95, and pH 8.52. The 0.2 M pH 3.89 buffer solution was prepared from CH_3_COOH and CH_3_COONa·3H_2_O, while 0.2 M pH 6.95 buffer was prepared from NaH_2_PO_4_·2H_2_O and Na_2_HPO_4_·2H_2_O. The 0.2 M pH 8.52 buffer was prepared similarly from NH_3_ and NH_4_Cl. The pH values of all buffers were measured immediately before use.

#### 2.4.2 Hydrolytic Degradation

The degradation was followed by hydrolytic degradation in the different pH buffers by monitoring the evolution of the weight and pH changes. Small segments of (0.5 × 0.5) cm^2^ of the chosen formulation and commercial PUR were dried at low temperatures (40–60) °C, weighed, and submerged in 20 mL of the different buffer solutions at room temperature in closed separated containers for the desired degradation times. One sample from each pH was removed every 2 weeks, dried at low temperatures (40–60) °C for at least a day to remove any traces of the buffer solution, and weighed. The pH values of the remaining solutions were measured immediately after the removal of the samples. All measurements were carried out in duplicate.

## 3 Results and Discussion

### 3.1 Experimental Design

#### 3.1.1 Design Using Microstructure (SEM)

**Figure 1:**
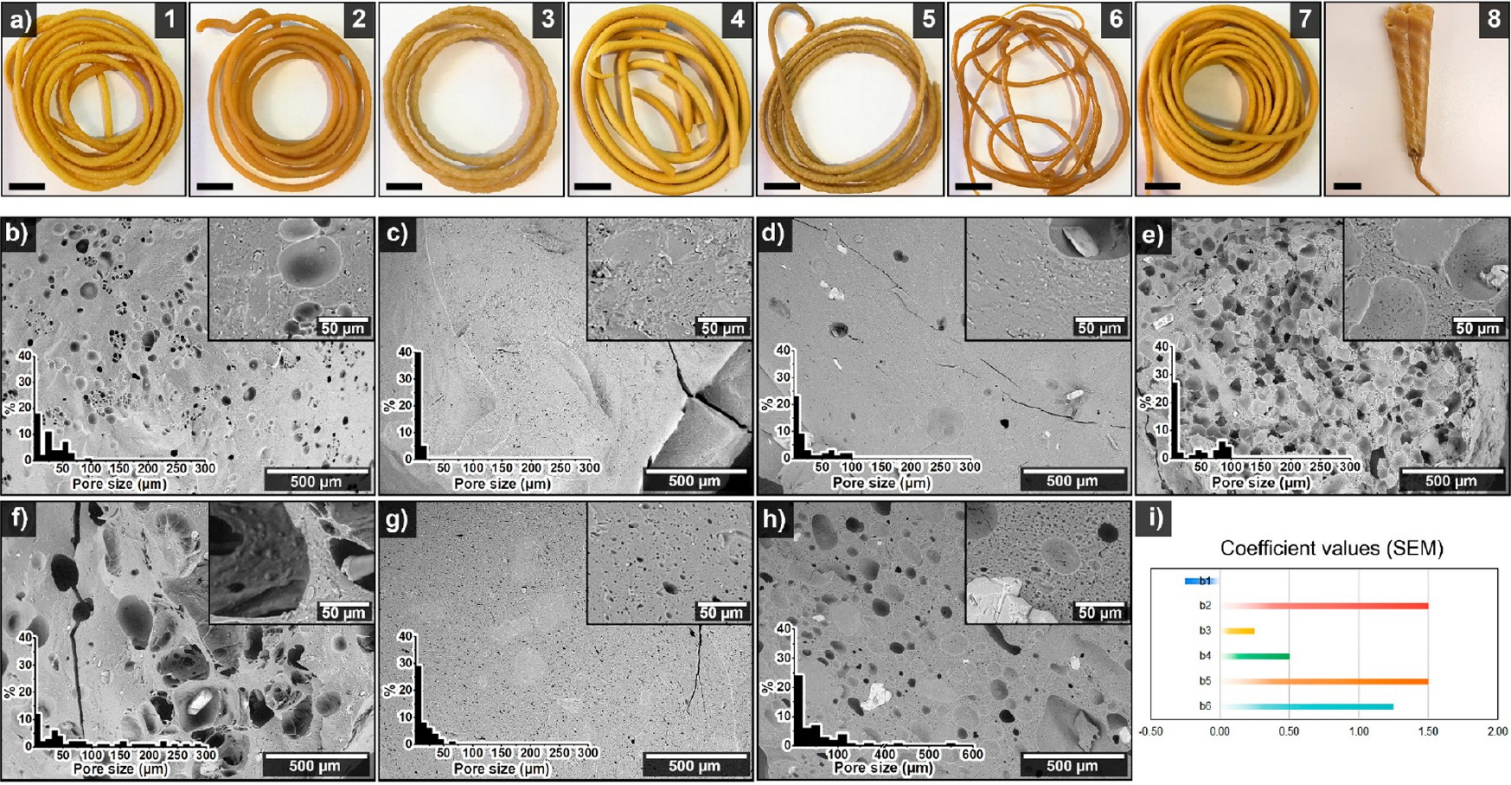
Visual aspect of the extrudates according to the experimental design parameters from the Hadamard matrix, Samples 1–8 (a, left to right, respectively). The scale bar in part a is 2 cm. SEM images of the extrudate cross-sections (Samples 1–7, b–h, respectively). Sample 8 is not shown because it was not possible to extrude in filaments. The inset in the cross-sectional images is a high magnification of the extrudates and the pore size distribution histogram. The resulting coefficient values from the Hadamard matrix when using the porosity as response parameter (i).

Figure 1a (1) shows the extruded protein-based filaments using the 8 formulations derived from the Hadamard matrix (Tables 1 and 2). All formulations were extrudable except for sample 8 (25WG/75Z/30Gly, extruded at 80 °C and 30 rpm), which became stuck in the barrel, as shown in Figure 1a. The trend revealed that the formulations with low glycerol (30 wt %), no water, and/or high gluten content had rough surfaces and showed high pressure during the process (compare samples 3 and 5, Figure 1a). The samples with a high zein content (sample 6) resulted in smoother surfaces despite the low glycerol/water content than that with a high WG content (sample 5).

The cryo-fractured cross sections of the filaments show that the sample having more promising pore size distribution is sample 4, with homogeneous and rounded medium-sized pores of 50–100 μm diameter with microsized porous on the cell walls of <10 μm (Figure 1e – 75Z/25W/60Gly/5SB, 80 °C, 60 rpm). The filaments from samples 1 and 7 also showed medium-sized pores on the cross-section. However, the inset in Figure 1b shows that sample 1 (25Z/75WG/60Gly/5MQ, 80 °C, 60 rpm) had denser cell walls with less porosity than sample 4 (Figure 1e). Also, sample 7 (75Z/25WG/60Gly/5SB/5MQ, 100 °C, 30 rpm) presented more microsized pores on the cell walls than sample 1 (Figure 1h and b, respectively). Sample 5 (high WG content) resulted in a porous but nonhomogenous microstructure with large, medium, and microsized pores (Figure 1f). The result suggests that combining water with sodium bicarbonate and high zein content favors the formation of homogeneous filaments with microsized and medium-sized pores toward bimodal pore size distribution. The property is interesting for porous absorbents mimicking the structure of commercially used polyurethane foams in single-use sanitary articles.^22,23^

Figure 1i shows the influence of the different formulation and processing parameters on the coefficient values from the Hadamard matrix when porosity is used as a response. It is important to remark that it is difficult to calculate a reliable statistical interval when using porosity as a response, as it relies on scoring criteria based on visual observations (Table S2). However, some conclusions can be drawn from the parameters, resulting in the highest response (b*i*) and correlated with the microstructure of the filaments. Accordingly, the amount of glycerol in the protein formulations (X2, response b2) and the extrusion speed (X5, response b3) were the two most influential factors on the porosity of the material (highest *Y*, Figure 1i). The sodium bicarbonate content (X6, response b6) represented the second-highest response toward increasing the formation of homogeneous porosity in the material. Although the effect of glycerol on the porosity of extruded biomaterials is scarce, it is known that glycerol can considerably impact the microstructure of biobased matrixes.^24−26^ The statistical analysis agrees with the resulting microstructures showing bimodal and homogeneous porosities when plasticizers (glycerol or glycerol/water) are combined with sodium bicarbonate. Thus, the glycerol content and extrusion speed on the porous microstructure were studied on samples combining 75Z/25WG/5SB/5MQ (extruded at 100 °C), which resulted in the most promising mimicking structures for sanitary absorbents and smooth/homogeneous protein-based filaments.

##### 3.1.1.1 Effect of the Amount of Glycerol

**Figure 2:**
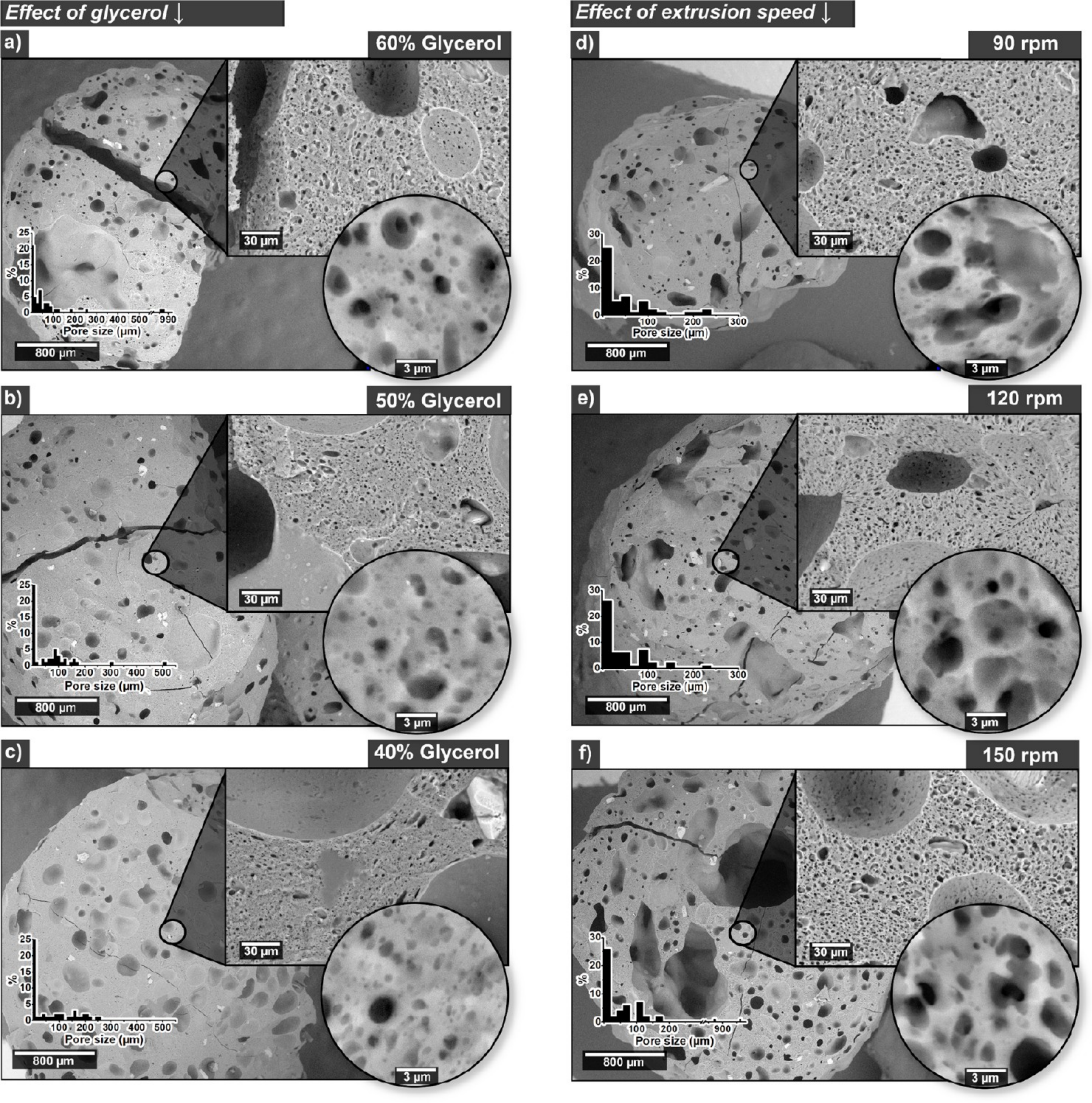
SEM images of the 75Z/25WG/5SB/5MQ cross sections (extruded at 100 °C and 60 rpm) with varying glycerol content (60, 50, and 40 wt %, a–c, respectively) and 75Z/25WG/60Gly/5SB/5MQ cross sections (extruded at 100 °C) with varying extrusion speeds (90, 120, and 150 rpm, d–f, respectively). The glycerol content is the total protein content. The inset in the cross-section images is a high magnification of the extrudates and the respective pore size distribution histogram.

Figure 2a shows that adding a high amount of glycerol (60 wt %) contributes to large macroporosity in the material with regular medium-sized pores of 50–150 μm. The cell walls of the 75Z/25WG/5SB/5MQ sample with 60 wt % glycerol showed a high content of micropores ranging from <1 to 15 μm (Figure 2a and Figure S3). Reducing the glycerol content to 50 wt % also reduced the presence of macropores while sharpening the micropore size distribution between <1 and 5 μm (Figure 2b and Figure S3). The lowest glycerol content tested here (40 wt %) revealed a broader medium-sized pore distribution, between 100 to 300 μm with similar behavior on the micropore size distribution on the cell walls as for 50 wt % glycerol (Figure 2c and Figure S3). The results indicate that high glycerol content increases the porosity, pore sizes, and interconnectivity, which are attractive parameters for large absorbent structures relying on capillary actions.^23^ Despite glycerol being a large coproduct from the transesterification process for biodiesel manufacturing,^27^ it is also water-soluble as it could jeopardize the material’s performance when exposed to liquid during absorption.^17^ We estimated that an optimal glycerol content to preserve regular pore size distribution at large and microscale for engineering production of these biomaterials is 50–60 wt %. Thus, 60 wt % glycerol content on the 75Z/25WG/5SB/5MQ system is used in the following section to evaluate the effect of different extrusion speeds on the microstructure of the materials. For producing the final prototypes, 50 wt % glycerol is used as a typical plasticizer content in protein manufacturing.^28^

##### 3.1.1.2 Effect of the Extrusion Speed

Figure 2d–f shows that high extrusion speeds promote macropore formation (above 500 μm) in the material (compare Figure 2d with Figure 2f). The results show that the extrusion speed does not ultimately have a considerable impact on the size of the small porosities located in the materials’ cell walls (below 20 μm) and on the medium-size porosity size distribution (see insets in Figure 2d,e and Figure S4). The formation of large pores in the materials with the highest extrusion speed tested here (150 rpm) could be related to the high shear forces and pressures at the exit of the extruder, which increases the abrupt expansion of the CO_2_ and water vapor coming from the degradation of sodium bicarbonate and water, respectively. Excessively large pores are detrimental to the absorption and homogeneity of the material, as the capillary forces retaining the liquid in the porous structure are low. High extrusion speeds are preferred from an engineering perspective as the production of the material is increased. Thus, it is suggested to use 90 rpm as the upper extrusion limit, with 60 rpm as an optimal extrusion speed to improve the material’s porosity (Figure 1e). Relatively longer residence time by the formulations within the extrusion barrel can increase the amount of CO_2_ generated by the foaming agent, as the temperature used is lower than the maximum degradation peak for the bicarbonate (*ca.* 120 °C).^17,20^

#### 3.1.2 Design Using Density

**Figure 3:**
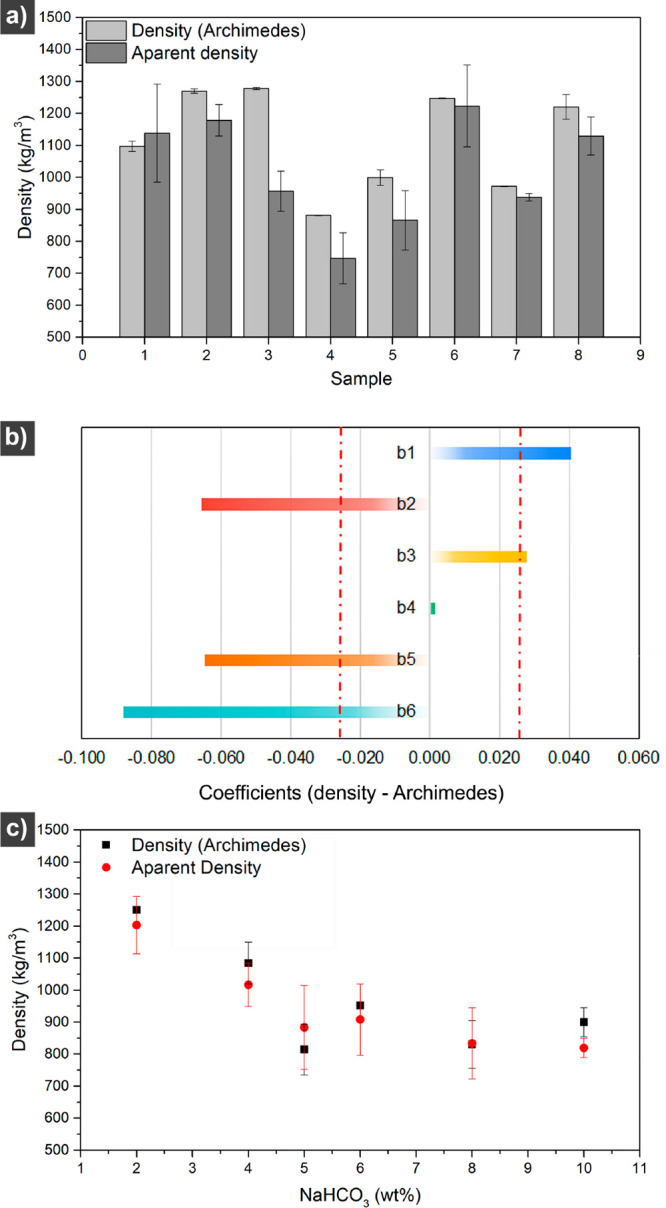
Density (Archimedes) and apparent density of the Sample formulations (8) from the Hadamard matrix (a). The resulting coefficient values from the Hadamard matrix when using the density as a response parameter (b). The density of the material with varying sodium bicarbonate content with respect to the protein content (c).

Figure 3a shows the density values (Archimedes and apparent densities) for the samples from the experimental design. Sample 4 had the lowest density of *ca.* 800 kg/m^3^, followed by Samples 5 and 7, while samples 2, 3, and 6 had the highest density of *ca.* 1300 kg/m^3^. The result agrees with the microstructures of the samples shown in Figure 1. The Archimedes and apparent density resulted in similar values between each sample except for sample 3 (75Z/25WG/30Gly/5SB/5MQ extruded at 80 °C and 30 rpm), which is related to the irregular shape of the filament, making the apparent density inaccurate.

Figure 3b shows the Hadamard matrix response values according to each material’s densities (Archimedes). In the case of the density, the goal is to minimize it, which is why the graph trend is the opposite when using the microstructure to maximize porosity (Figure 1i). As the density results from an average and represents a precise experimental value, we can calculate the confidence interval of the coefficients (*bi*). We obtain *bi* ± = 0.024 (red lines in Figure 3b). Only factor 4, which corresponds to the extrusion temperature, does not fall within the confidence interval. Therefore, the extrusion temperature can be placed at the −1 or +1 level without significantly impacting the material. The result can be attributed to the small difference between the two temperatures studied (80 and 100 °C). The response values within the confident interval show that the amount of sodium bicarbonate (b6) becomes the most influential factor when considering density as the statistical factor, followed by the amount of glycerol (b2) and then the extrusion speed (b5) (Figure 3b). The experimental design results confirm that the two most important factors in producing highly porous (lowest density) protein bioblend structures are glycerol and the extrusion speed. The sodium bicarbonate content was then varied to evaluate its effect on minimizing the density of the material according to the Hadamard matrix results (Figure 3b).

##### 3.1.2.1 Effect of the Sodium Bicarbonate Content

Figure 3c shows that the increase in the sodium bicarbonate content results in a gradual decrease in the density of the material (Archimedes and apparent density). The results agree with the experimental design showing bicarbonate as a highly influencing factor in the density of the material. The density was decreased by *ca.* 45%, adding solely 5 wt % NaHCO_3_ to the material while still producing stable extrudates with homogeneous porosity. Therefore, 5 wt % of the bicarbonate was selected as the optimal amount to decrease the density of the protein biohybrid porous extrudates.

The water content was also tested as our previous work show that the presence of water can also act as a protein plasticizer and as a foaming agent of gluten materials.^18,20,22^ The use of water as a plasticizer and foaming agent was shown to be detrimental to the formation of medium-size and microsize pores (see Figure S5). Thus, the results show that the amount of water should be kept within 0 to 5 wt % to avoid collapse of the medium-size pores.

All in all, to maximize the porosity of the material, the recipes 75Z/25WG/5SB/5MQ with glycerol content of 40, 50, and 60 wt % and 75Z/25WG/50Gly/5SB were selected for their evaluation as absorbents in single-use sanitary articles and their production in pilot-scale equipment. A sample of only zein, gluten, and glycerol was used as a reference (75Z/25WG/50Gly).

### 3.2 Absorption Properties and Assembly as an Absorpbent Item

**Figure 4:**
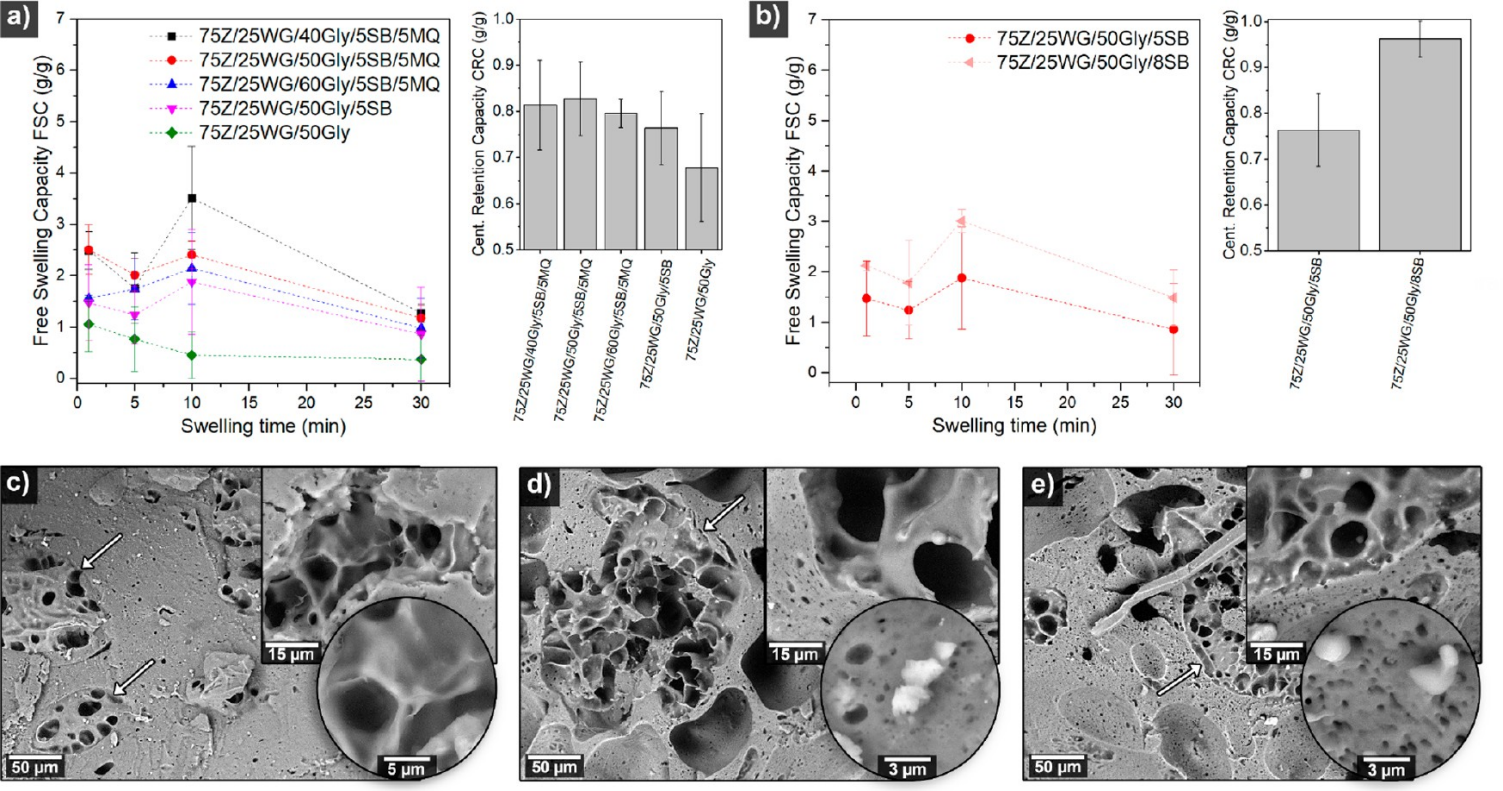
Free swelling capacity (FSC) of the extrudate recipes derived from the experimental study with varying glycerol and water content (a). FSC of the extrudate recipes with varying sodium bicarbonate content (b). The inset in a and b shows the materials’ centrifuge retention capacity (CRC). SEM micrographs of the cross-section of the extrudate after 30 min swelling in saline solution and further lyophilization: 75Z/25WG/50Gly, 75Z/25WG/50Gly/5SB, 75Z/25WG/50Gly/5SB/5MQ, c–e, respectively. The insets in the SEM micrographs show high-magnification images, and the arrows point at the highly swollen domains.

Figure 4a shows that the saline free swelling capacity (FSC) of 75Z/25WG/50Gly/5SB/5MQ the material reached up to 3 g/g within the first minute, followed by a gradual decrease in the FSC. The reduction in the swelling in all samples corresponds to extensive mass loss due to the high solubility of glycerol as demonstrated in previous works.^17,20^ The porous materials reached up to 4 times more water uptake as compared to the reference nonporous material (see 75Z/25WG/50Gly Figure 4a). The centrifuge retention capacity (CRC) of all porous materials was *ca.* 0.8 g/g, slightly higher than the nonporous reference material (Figure 4a inset). Figure 4b shows the effect of the material porosity on the FSC, increasing from 2 to 3 g/g when increasing the NaHCO_3_ content from 5 to 8 wt %, respectively. The inset in Figure 4b also shows that the CRC increases from 0.75 to 0.95 g/g when using a higher bicarbonate content. Future strategies for developing porous absorbent materials should focus on improving the liquid absorption capacity, for instance, grinding the material into a fine porous powder and/or increasing the pore interconnectivity and permeability (more information discussed below).

The cross-section of the lyophilized 75Z/25WG/50Gly material after 24 h of 0.9 wt % NaCl swelling shows regions where new porosity is formed (see Figure 4c). Similarly, 75Z/25WG/50Gly/5SB/5MQ and 75Z/25WG/50Gly/5SB also presented newly developed porous regions after the saline swelling, which does not correspond to the original pore structures observed in the material (see Figure 4d and e, respectively). The interface between the porous domains and the matrix was continuous, and some NaCl crystals were observed embedded on the thin cell walls (see Figure 4d and e, insets). Samples of pure zein with 50 wt % glycerol and 5 wt % SB were also extruded (Zn/50Gly/5SB), swelled in saline solution, and lyophilized (see Figure S6). The reference extrudate Zn/50Gly/5SB collapsed upon cooling while still keeping a porous structure, indicating that gluten also provides structural stability to the material (Figure S6a). After swelling and lyophilization, the cross-section of the material reveals a whiter surface with large pores formed, indicating that the saline solution had penetrated only the surface of the extrudates (Figure S6b). At the same time, no new porous domains were observed in this sample before and after swelling as for 75Z/25WG/50Gly/5SB/5MQ and 75Z/25WG/50Gly/5SB (see Figure S6b and Figure 4d,e, respectively). Thus, it is suggested that the newly developed porous regions in 75Z/25WG/50Gly/5SB/5MQ and 75Z/25WG/50Gly/5SB are WG domains within the zein matrix, as WG has a higher water affinity than the nonwater-soluble zein protein.^18,19,29,30^ The system resembles the lyophilized sections of semiIPN systems based on polysaccharides biohybrids hydrogels.^31,32^ The absorption under load (AUL) against saline solution was tested on the 75Z/25WG/50Gly/5SB/5MQ sample and resulted in 0.6 ± 0.1 g/g (pressure used: 0.25 psi). The result shows the importance of testing the material on user conditions for their future utilization as absorbent materials in hygiene articles.

**Figure 5:**
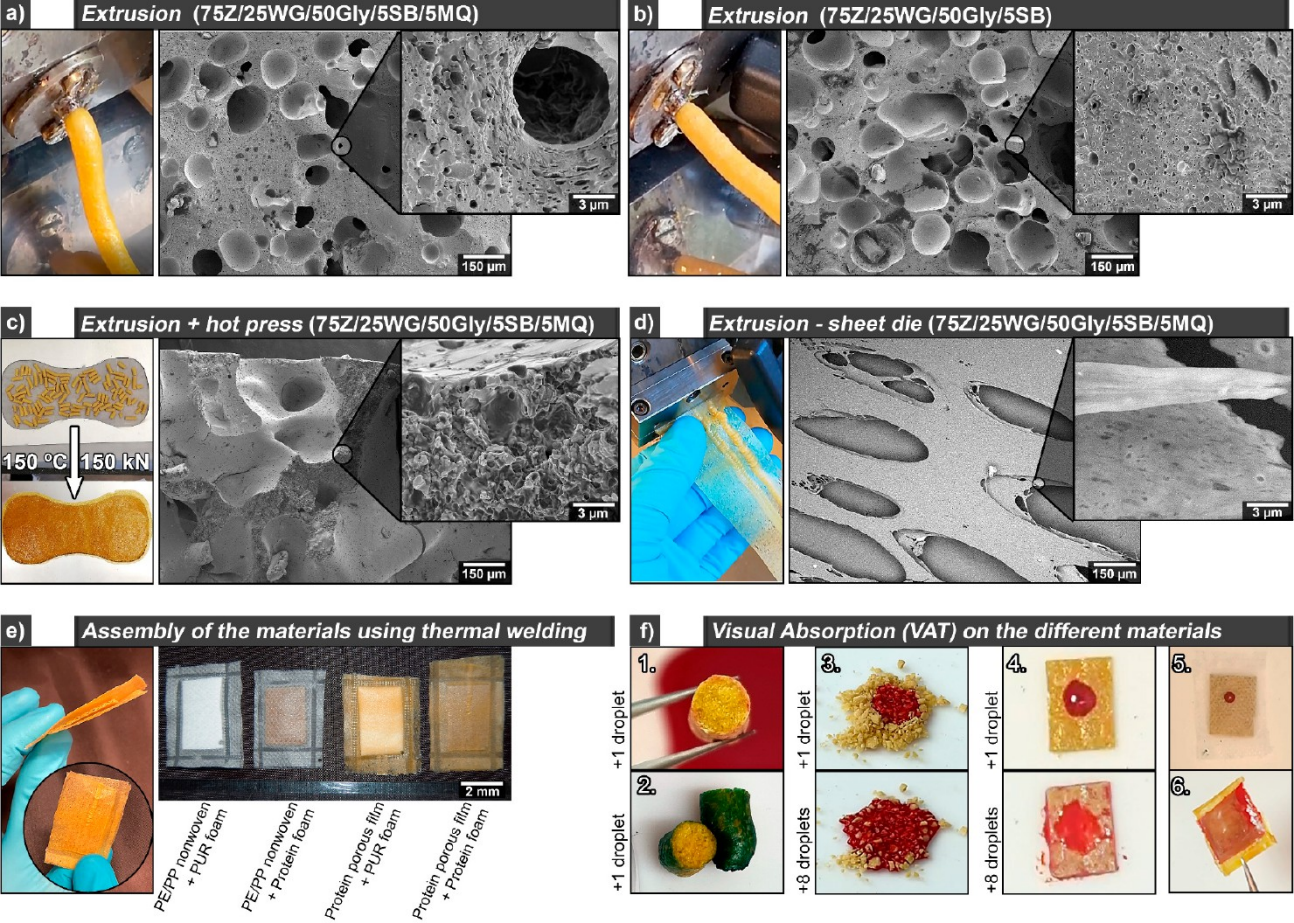
Extruded 75Z/25WG/50Gly/5SB/5MQ (a) and 75Z/25WG/50Gly/5SB (b) using a cylindrical die and the respective cross-section. Compression molding of the pellets from the extrusion of 75Z/25WG/50Gly/5SB/5MQ using a pad mold and the respective cross-section (c). Extruded films of the 75Z/25WG/50Gly/5SB/5MQ using a sheet die and the respective surface under SEM (d). Thermal assembly of the extruded sheet-die containing the extruded + compression molded porous structures and different material combinations (e). Visual absorption capacity and spreading properties of the material in different shapes (f) as extruded (75Z/25WG/50Gly/5SB/5MQ and 75Z/25WG/50Gly/5SB, 1. and 2., respectively), extruded 75Z/25WG/50Gly/5SB/5MQ grounded into porous powders (3.), extruded + compressed protein pads (4.), assembly of the PE nonwoven film and extrusion + hotpressed protein pad (5.), assembly of extrusion-sheet die porous protein film + extrusion-sheet die solid protein film, encapsulating a porous extruded + compressed protein pad (6.). The visual absorption capacity in 2. is a saline solution with a blue die, and 3.–6. defibrinated sheep blood.

The 75Z/25WG/50Gly/5SB/5MQ and 75Z/25WG/50Gly/5SB were further explored to prepare functional porous absorbent sanitary materials. Figure 5a and b show the 75Z/25WG/50Gly/5SB/5MQ and 75Z/25WG/50Gly/5SB filaments during the extrusion process on the twin screw extruder (circular die, 100 °C, 60 rpm). The materials were visually highly homogeneous with constant flow, an estimated 0.5 kg/h production rate, and an expansion ratio of 1.4 ± 0.2. The microstructure also showed homogeneous medium and microsized pores distributed in the entire cross-section with more deformed pores in the proximity of the surface in both 75Z/25WG/50Gly/5SB/5MQ and 75Z/25WG/50Gly/5SB (Figure S7 and Figure S8, respectively). The surface of the extruded filaments was also denser than the matrix and, coupled with the deformed pores closed to the surface, shows the high pressure developed from the inner part of the material once it exited the extruder (Figure S7 and S8, insets). The formulations 75Z/25WG/50Gly/5SB/5MQ and the reference 75Z/25WG/50Gly were pilot-tested in a single-screw Brabender resulting in homogeneous extruded filaments resembling those extruded in the microcompounder and with production rates of *ca*. 1.5 kg/h (Supplementary Vdeo S1). The results demonstrate for the first time the possibility of producing the suggested protein biohybrid formulations with scalable properties for the future mass production of these materials as single-use functional absorbent materials.

Figure 5c shows the 75Z/25WG/50Gly/5SB/5MQ extruded filaments chopped into pellets and successfully converted to a porous pad prototype. The cross-section of the hot-pressed pad revealed large *ca*. 500 μm pores while displaying microsized pores on the cell walls (Figure 5c, inset, and Figure S9). The pad showed a flexibility comparable to that of a commercial pad based on polyurethane (PUR) foam, shown in Figure S10. The formulation was also extruded into a porous film by using a film die with a slit height of 0.2 mm (see Figure 5d). Figure 5d (inset) shows the microstructure of the porous film with elongated pores of *ca*. 300 μm length on the longest axis. The porous hot-pressed pad was encapsulated between the porous film and sealed by using pulse welding, as shown in Figure 5e. A PUR foam from a sanitary pad was also encapsulated on a PE/PP nonwoven film as a reference prototype and sealed as the full protein biohybrid prototype and combinations thereof. The different varieties of materials for prototyping are shown in Figure 5e, with an example of the welding line from a protein porous film and PUR foam shown in Figure S11.

The absorption of extruded 75Z/25WG/50Gly/5SB and 75Z/25WG/50Gly/5SB/5MQ was below 0.5 g/g (both blood and saline solution). The low absorption accounted for the saline/blood’s inability to penetrate the porous structure due to a solid skin formed in the material after extrusion (Figures 5f–1. and 2. and Figures S7 and S8). The effect of the liquid not penetrating the outer shell of the extrudates is shown in Figure 5f–2., using a blue die in the saline solution. The low absorption values of the porous extruded filaments in the VAT test were improved by cryogenic grinding of the extruded material into porous particles. The grinding preserved the porous nature of the originally extruded filaments (Figure S12) and allowed for an increase in the liquid spreading and VAT to 4 g/g (Figure 5f–4.). On the contrary, the cryogenic fractured nonporous filaments (75Z/25WG/50Gly) resulted in a blood VAT of only 0.4 g/g. The same trend was observed when grinding the filaments and performing the VAT using 0.9 wt % NaCl solution, indicating that grinding the porous filaments is a promising alternative to increase the access of the liquid within the porous particle structure (Figure S13 and Figure S14). No porosity was formed after swelling in saline solution and further lyophilization of the reference 75Z/25WG/50Gly, agreeing with the low values of FSC and VAT previously observed (Figure 4c and Figure S15).

**Figure 6:**
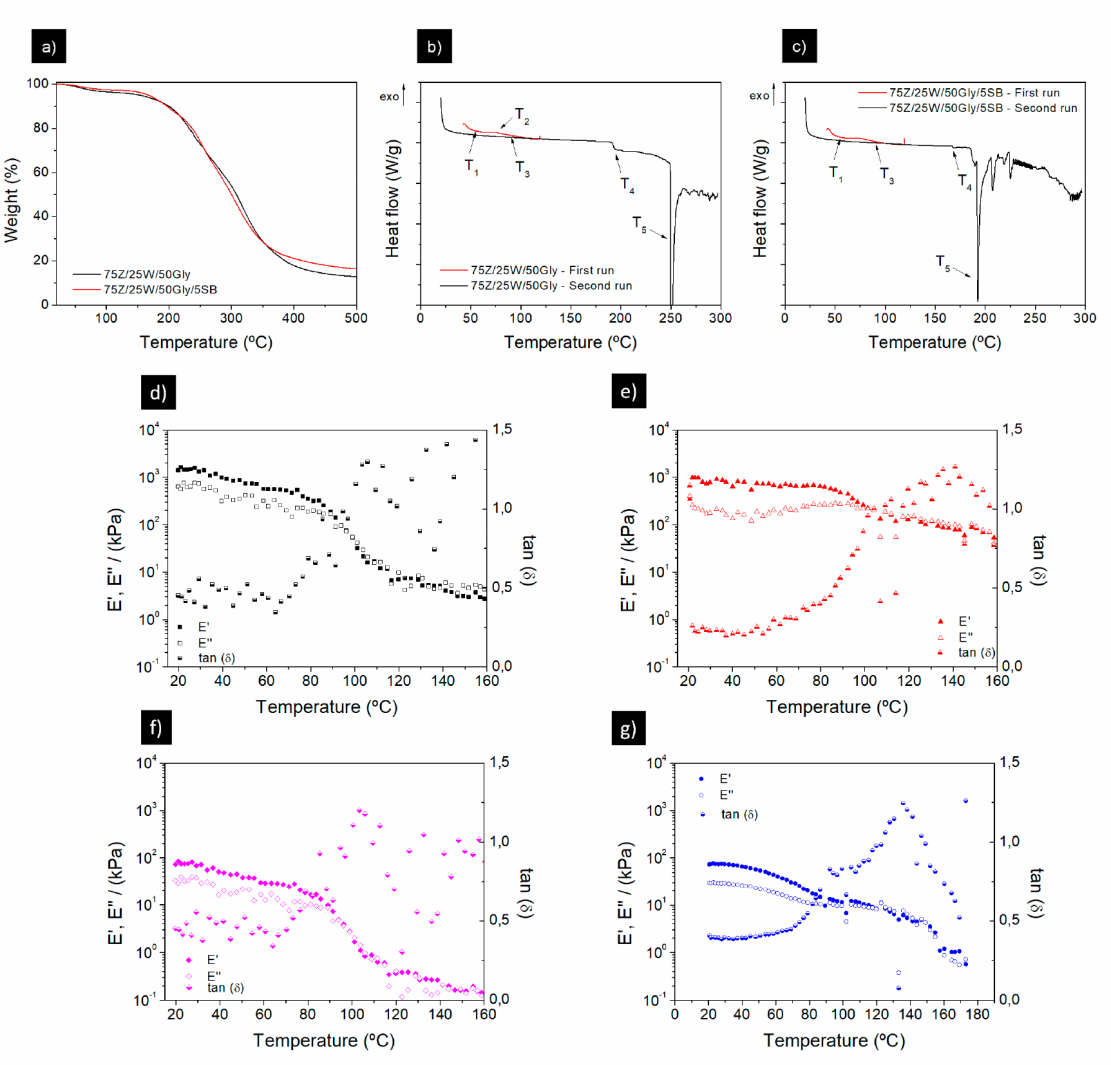
Thermogravimetric analyses (a) and differential scanning calorimetry of zein-gluten (b) and zein-gluten-bicarbonate (c) blends without processing. Temperature ramps of zein-gluten (d), zein-gluten-bicarbonate (e), zein-gluten-water (f), and zein-gluten-bicarbonate-water blends (g). All blends contain 50 wt % glycerol.

Figure 5f–4. shows that the blood spread through the extruded + hot press 75Z/25WG/50Gly/5SB sample and reached a VAT of 4.2 ± 0.7 g/g. The higher VAT compared to the extruded porous 75Z/25WG/50Gly/5SB is due to the open pore microstructure of the material, even on the external layers (Figure S9), allowing the viscous blood to penetrate the network efficiently, as seen in Figure S16. This sample’s rapid and high blood absorption capacity is illustrated in Supplementary Video S2. The combination of the PE/PP nonwoven film encapsulating the protein blend foam (75Z/25WG/50Gly/5SB) did not show adequate spreading of the blood droplet; the droplet stayed at the surface of the material and only penetrated when pressed (Figure 5f–5.). The same behavior was observed in a commercial sanitary product (Figure S17). Using the extrusion-sheet die protein blend nonwoven (75Z/25WG/50Gly/5SB/5MQ, Figure 5d) encapsulating a synthetic PUR foam resulted in a VAT of 16.8 g/g (Figure S18). Here, the droplet was absorbed into the network without the need to press the droplet against the material. Encapsulating the protein foam between the extruded + sheet die porous film had a low VAT of 0.7 g/g. The low absorption accounted for the blood leaked from the porous protein film at the back side of the prototype, which set the test’s end-point according to standards (Figure S17). A solid protein film (75Z/25WG/50Gly) was extruded with the sheet die and used on the back side of the prototype. The blood was effectively encapsulated within the prototype, resulting in a VAT of 1.9 g/g (Figures 5f–6.). Figure S19 shows the screenshots from the contact angle measurement of the 75Z/25WG/50Gly/5SB/5MQ (extruded + hot pressed), demonstrating the high hydrophilicity/wettability of the materials, which generally adsorbed the deposited droplet within less than 2 s (see Supplementary Video S3). The absorption and facile way of assembling the protein blend layers demonstrated the potential to produce different material shapes, which have a key role in the assembly of modern disposable sanitary pads. Further, the reported porous microstructure and liquid absorption capacity of these protein-based porous materials can have a role in novel applications such as in biomedical engineering, especially in the development of 3D scaffolds or biodegradable dressings.^33,34^Table S3 shows that the estimated product price is 7.33 USD/kg of material, which takes into consideration 20% of production and maintenance cost. The price was estimated on the formulation using the most expensive reagents here (*i.e.*, 75Z/25WG/50Gly/5SB) and was of similar ranges to some commercially available synthetic superabsorbents, as shown in Table S4.

Future studies should focus on evaluating the stability of the products during long-term storage (1 year). Here, changes in the liquid absorption simulating storage conditions and/or plasticizer migration over time (as previously reported in protein-based materials)^35^ should be considered to assess the product’s quality further.

### 3.3 Processing Properties of the Materials

The molecular and thermal characteristics of the blends were assessed by their thermal and rheological properties. Figure 6a shows that blends (with and without SB) exhibited the same TGA profile, with a first weight drop at 100 °C to 93–95%. This first drop gave information about the moisture content of the samples, being in the range of 5–7%. The sharpest weight drop occurs between 250 and 300 °C down to 30%, related to the degradation of most of the components (proteins, lipids, *etc*.). Finally, from 450 °C onward, the inorganic components of the blend (minerals) remained lower than 20% for both samples. The profiles agree with previous results on biopolymers.^36^ Here, the TGA profile is better defined and smoother for the sample with sodium bicarbonate, indicating a greater thermal stability.

Figure 6b and c show the DSC profiles of the protein blends before processing. Both profiles exhibited the same four thermal transitions, starting for the physical aging at *ca*. 60 °C,^37^ followed by the glass transition region (*T*_g_)^38^ of the blend between 90 and 95 °C (Table 3). Finally, there are two thermal transitions at higher temperatures due to denaturation and melting of the proteins. It is worth mentioning that the addition of the bicarbonate induced two different effects: the thermal transitions were shifted toward lower temperatures, and it favored the homogeneity of the mixtures since the transition at 78 °C, ascribed to gluten thermosetting,^39^ did not appear (Figure 6c).

**Table 3:** Thermal Parameters Obtained for the Zein-Gluten and Zein-Gluten-Bicarbonate Blends

sample		temperature (°C)	signal
Blend 75Z/25W/50Gly	T1	58	Physical Aging
	T2	78	Thermosetting of gluten
	T3	94	*T*_g_
	T4	192	Denaturation
	T5	250	Melting
Blend75Z/25W/50Gly/5SB	T1	56	Physical Aging
	T2	-	-
	T3	90	*T*_g_
	T4	168	Denaturation
	T5	194	Melting

Figure 6d–g show the temperature ramps of the blends produced with and without sodium bicarbonate and water. Both mixtures showed a similar trend, *i.e.*, decreasing *E*′ values up to an inflection point. This inflection point corresponds to the protein’s *T*_g_ and the maximum value observed in tan δ. As shown in Table 4, the *T*_g_ ranges between 95 and 105 °C, which matches the values obtained for the DSC analyses (Figure 6b). Again, the thermal transitions appear at earlier temperatures with the addition of bicarbonate to the blend (whether it has water or not).

**Table 4:** Parameters Obtained from the Dynamic Compression Tests (Elastic Modulus at 1.0 Hz, *E*′_1_; Loss Tangent at 1.0 Hz, tan (δ)_1_; and Critical Strain) and Glass Transition Temperature (*T*_g_) of Zein-Gluten and Zein-Gluten-Bicarbonate Blends

sample		*T*_g_ (°C)	critical strain (%)	*E*′_1_ (kPa)	tan (δ)_1_
75Z/25W/50Gly	20 °C	104 ± 7	0.141 ± 0.024	620 ± 197	0.31 ± 0.01
	80 °C		0.182 ± 0.011	154 ± 28	1.01 ± 0.04
	100 °C		0.350 ± 0.193	219 ± 32	0.87 ± 0.04
	140 °C		-	-	-
75Z/25W/50Gly/5SB	20 °C	96 ± 7	0.096 ± 0.030	1311 ± 105	0.26 ± 0.01
	80 °C		0.386 ± 0.032	281 ± 216	0.91 ± 0.03
	100 °C		0.048 ± 0.012	107 ± 13	0.61 ± 0.06
	140 °C		0.081 ± 0.045	41 ± 35	0.87 ± 0.08
75Z/25W/50Gly/5MQ	20 °C	100 ± 4	0.215 ± 0.027	130 ± 41	0.32 ± 0.02
	80 °C		0.256 ± 0.021	69 ± 23	0.45 ± 0.03
	100 °C		0.393 ± 0.074	7 ± 2	0.88 ± 0.02
	140 °C		-	-	-
75Z/25W/50Gly/5SB/5MQ	20 °C	87 ± 6	0.146 ± 0.036	69 ± 14	0.50 ± 0.04
	80 °C		0.486 ± 0.103	47 ± 19	0.73 ± 0.07
	100 °C		0.077 ± 0.014	54 ± 8	0.85 ± 0.03
	140 °C		-	-	-

The presence of water or bicarbonate in the protein blends generates differences even if they maintain the same thermal profile. The water retains the rheological shape but results in lower moduli values. This may be due to the plasticizing effect of water, which generates a more fluid system. On the contrary, the presence of bicarbonate, irrespectively with or without water (Figure 6g and e), did not show a steep decrease of moduli in the inflection zone compared to those without SB (Figure 6d and f). Therefore, higher moduli values are achieved after inflection in the systems with bicarbonate. This effect could be due to the greater presence of pores in the material when the SB is incorporated, alleviating the moduli decrease at higher temperatures.

Strain and frequency sweep tests were also performed on the blends at different temperatures. The chosen temperatures were selected from the temperature ramps shown in Figure 6d, *i.e.*, 20 °C as the starting point, 80 °C before the inflection point, 100 °C after the *T*_g_, and 140 °C once the glass transition and stabilization were achieved (Figure S20). According to the critical strain values shown in Table 4, more deformable systems are obtained at temperatures close to the *T*_g_. In contrast, the deformability decreased overcoming the glass transition of the mixture; *i.e.*, the critical strain of 75Z/25W/50Gly/5SB blend decreased from 0.048 to 0.386 (80 °C) to 0.048% (100 °C). Nevertheless, this effect is only observable when bicarbonate is incorporated (75Z/25W/50Gly/5SB and 75Z/25W/50Gly/5SB/5MQ blends) because temperatures could be measured after the inflection zone. The frequency sweep tests (Figure S20) showed a similar profile for the blends up to 100 °C, with a slight increase in the *E*′ values at higher frequencies. The solid character decreased by increasing the temperature, as shown by the proximity of the *E*′ and *E*″ values at 80 and 100 °C due to the glass transition and change from a solid state to a soft rubbery state.^40^ These changes can be better seen by following the tan (δ) values (Table 4), which increase when the frequency sweep is conducted at higher temperatures. The frequency sweep test at 140 °C could only be carried out for the 75Z/25W/50Gly/5SB blend, suggesting a more thermal stable combination than the other systems. Nevertheless, its profile was unstable and did not show the same trend as lower temperatures.

**Figure 7:**
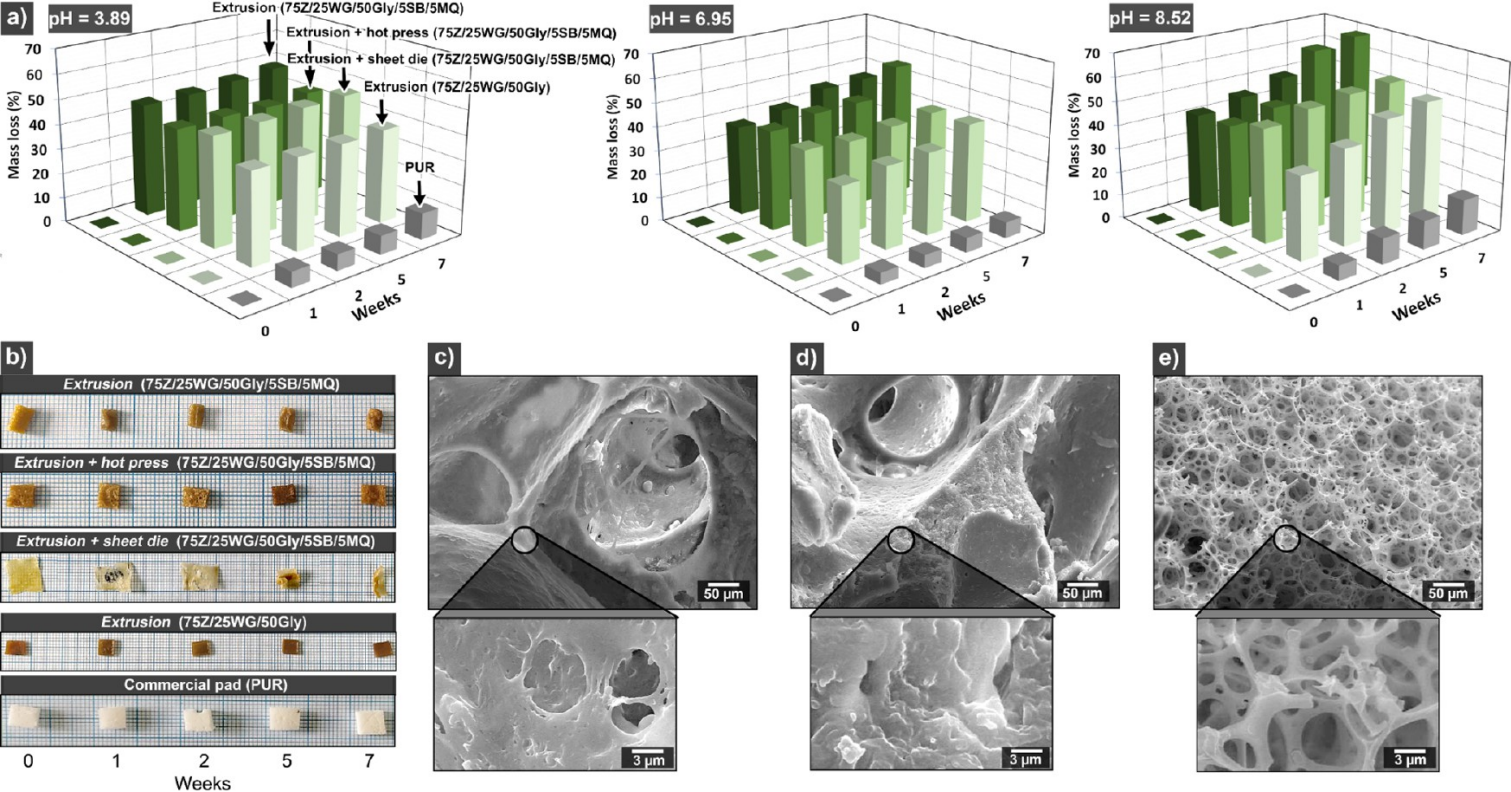
Mass loss of the different protein blends and commercial reference products with time during the hydrolytic degradation test in acidic (pH = 3.89), neutral (pH = 6.95), and alkaline medium (pH = 8.52) (a). The visual aspect of the materials on the alkaline medium is shown in (b). The large square box in the figure represents 1 cm. Internal morphology of the extruded 75Z/25WG/50Gly/5SB/5MQ, extruded + hot pressed 75Z/25WG/50Gly/5SB/5MQ, and commercial PUR sample after 7 weeks exposure in alkaline degradation, c–e, respectively.

The thermal and rheological tests allow us to determine the operating temperatures of these systems. Maintaining a temperature where the material can flow correctly is important during extrusion. Therefore, the selected processing temperature range is where the prepared mixtures have a rubbery state (80–100 °C). However, the small difference found between both modules (*E*′ and *E*″) allows the material to flow correctly at lower temperatures, allowing a more consolidated final material to be obtained (*E*′ > *E*″). This is also why it was possible to obtain extrudates at a lower temperature (30–60 °C) during this work.

Regarding pressed pads, the high temperatures allow for keeping the samples’ rubbery state and adequate mold filling. However, a curing temperature higher than *T*_g_ is necessary for bioplastic thermosetting due to denaturation of the proteins. In these systems, as occurred during extrusion, the small difference between *E*′ and *E*″ allows using a single temperature to fill the mold and cure the blends simultaneously, which must be between the *T*_g_ and the denaturation temperature. The suggested processing temperatures are 104–192, 96–168, 100–192, and 87–168 °C for 75Z/25W/50Gly, 75Z/25W/50Gly/5SB, 75Z/25W/50Gly/5MQ, and 75Z/25W/50Gly/5SB/5MQ systems, respectively.

### 3.5 Hydrolytic Degradation of the Materials

Figure 7a shows the mass loss of the different samples under hydrolytic degradation at acidic (pH = 3.89), neutral (pH = 6.95), and alkaline medium (pH = 8.52). All protein-blend products resulted in more than 40% hydrolytic degradation at 1 week, irrespective of the pH of the medium. The initial high mass loss corresponds to large glycerol loss in the aqueous solution and hydrolytically degraded material. On the contrary, the commercial polyurethane pad reference (PUR) only showed a maximum of 15% mass loss after 7 weeks in alkaline media, which was the most aggressive degradation buffer (Figure 7a). The mass loss for the PUR material is associated with surface erosion via esther bond hydrolysis promoted in alkaline conditions.^41^

The highest mass losses (%) of all materials were obtained in alkaline conditions (pH = 8.52), specifically for the extruded + hot pressed sample (75Z/25WG/50Gly/5SB/5MQ) with up to 70% in 7 weeks (Figure 7a). The lower degradation for the other protein-blend materials (*ca*. 60%) agrees with a less porous network, which decreases the surface available for microorganisms. The results also agree with previous studies suggesting that basic conditions favor peptide and amide bond hydrolyzation and removal/modification of functional groups.^42^

Figure 7b shows the macroscopic aspect of the different materials tested after removal from the hydrolytic alkaline medium and drying. Figure S21 shows the material after acidic and neutral hydrolytic treatment. The aspect/shape of the material in the acidic and neutral medium is maintained during the weeks without notable deformations or changes in coloration (Figure S21). On the contrary, Figure 7b shows that all protein-blend products showed a darker color or a structural collapse after 7 weeks of hydrolytic degradation, which corresponds well with these samples having the highest mass loss (Figure 7a). The PUR reference showed no visual signs of hydrolytic degradation, as shown in Figure 7b.

It is worth noticing that the hydrolytic media was not changed between the weeks, which allowed the following pH changes of the supernatant over time (Figure S22). For all the protein-blend products, the changes in pH were similar and resulted in the initial acidification of the medium due to glycerol lixiviation.^43^ Once again, the pH variation of the alkaline systems was the highest, which agrees with the high mass loss and is associated with possible deamidation of the proteins leaching acidic molecules such as carboxylic-terminal groups.^44^

Figure 7c and d show the microstructure of the extruded 75Z/25WG/50Gly/5SB/5MQ and extruded + hot pressed 75Z/25WG/50Gly/5SB/5MQ as representative samples after 7 weeks in alkaline degradation, respectively. Both showed larger pores than before the degradation, and the small pores were not visible (Figure 5a), demonstrating the material’s structural collapse after the extensive degradation in this medium. Figure 7e reveals that the PUR microstructure showed no signs of structural collapse.

## Conclusions

A porous material with high thermal processability even at low temperatures and scalable properties can be produced based on sole protein blends from industrial coproducts. The extrusion speed, plasticizer content, and amount of foaming agent resulted in the largest contribution to form continuous extrudates with homogeneous porosity. The continuous extrudates were only possible due to a synergy between gluten (large binder) and zein protein (lower molecular weight processing aid). The pores promote saline and blood absorption capacities, making them competitive for use as absorbent layers in disposable sanitary materials. The processing was performed at lower temperatures than those of commercial plastics in these articles. The readily assembling of the different layers using traditional polymer processing techniques (extrusion and compression) opens up their design as large, 100% protein-based scalable alternatives. Moreover, the protein-blend products showed up to 70% hydrolytic degradation in less than 5 weeks. The biodegradability and the unique design of 100% protein-based absorbents allow sanitary materials to be flushed in future industry. Overall, the alternatives contribute to a circular bioeconomy with protein raw materials from industrial costreams not competing with the food market, processed with low energy using industrial equipment (minimal resources investment), and degraded into innocuous molecules for nature.

## Data Availability

All data included in this study are available upon request from the corresponding author.

## Supporting Information Available

Visual aspect of the different premixtures (Table S1); scoring criteria for the extruded samples (Table S2); detailed cost estimation and final product price (Table S3); comparison of product prices (Table S4); hot-pressing scheme (Figure S1); AUL scheme (Figure S2); pore-size distribution of 75Z/25WG/5SB/5MQ samples at different glycerol content (Figure S3); pore-size distribution of 75Z/25WG/50Gly/5SB/5MQ at different extrusion speeds (Figure S4); SEM images of 75Z/25WG/50Gly/5SB at different water content (Figure S5); extruded zein samples and swollen samples with their SEM cross-section (Figure S6); SEM of the extruded 75Z/25WG/50Gly/5SB/5MQ filament (Figure S7); SEM of the extruded 75Z/25WG/50Gly/5SB filament (Figure S8); SEM of the hot-pressed 75Z/25WG/50Gly/5SB pad (Figure S9); prototype compared to commercial sanitary pad (Figure S10); porous film encapsulating a commercial PUR foam (Figure S11); SEM image of the 75Z/25WG/50Gly/5SB/5MQ extrudate after grounding (Figure S12); SEM image of the 75Z/25WG/50Gly/5SB extrudate after grounding and swelling (Figure S13); SEM image of the 75Z/25WG/50Gly/5SB/5MQ extrudate after grounding and swelling (Figure S14); SEM image of the 75Z/25WG/50Gly extrudate after grounding and swelling (Figure S15); defibrinated sheet blood retention of the material (Figure S16); commercial prototype defibrinated sheep blood adsorption behavior (Figure S17); natural prototype sheep blood adsorption behavior (Figure S18); contact angle screenshots (Figure S19); frequency sweep tests (Figure S20); images of visual aspect of the materials after hydrolytic degradation (Figure S21); pH changes of the hydrolytic media (Figure S22) (PDF)
Video S1: Video showing the extrusion of the porous protein blends in lab and pilot-scale (MP4)
Video S2: Video showing the VAT absorption in defibrinated sheep blood (MP4)
Video S3: Video showing the contact angle measurement on the extruded + hot pressed 75Z/25WG/50Gly/5SB/5MQ sample (MP4)
