# Supplementary material for: Porous Thermoformed Protein Bioblends as Degradable Absorbent Alternatives in Sanitary Materials
Source: ACS Appl Polym Mater. 2023 Aug 25;5(9):6976–89. doi: 10.1021/acsapm.3c01027 (PMC10497054; doi:10.1021/acsapm.3c01027)
Supplement: Supplementary file 1 — ap3c01027_si_001.pdf [file ap3c01027_si_001.pdf]

Supporting Information

Porous thermoformed protein bio-blends as degradable absorbent alternatives in sanitary materials

Agnès Jugé,[a] Jeannine Moreno-Villafranca,[b] Victor M. Perez-Puyana,[c] Mercedes Jiménez-Rosado,[c] Marcos Sabino,[b] and Antonio J. Capezza[a*]

[a]KTH Royal Institute of Technology, Department of Fibre and Polymer Technology, Polymeric Materials Division, School of Engineering Sciences in Chemistry, Biotechnology and Health, Stockholm 10044, Sweden.

[b]B5IDA Research Group Chemistry Department, Universidad Simón Bolívar USB, AP 89000, Caracas

[c]University of Seville, Department of Chemical Engineering, Seville 41012, Spain

Corresponding Author

Antonio J. Capezza ajcv@kth.se

Table S1. Visual aspect of the different pre-mixtures prepared to establish the initial material compositions for the experimental design study.

Sample	Wheat gluten	Zein protein	Glycerol	Observations	Visual aspect
**A**	1g		1 g (50%)	Consistency of highly wet pulp.	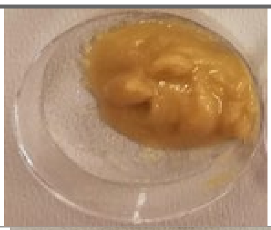
**B**	1.2 g		0.8 g (40%)	Consistency of wet pulp.	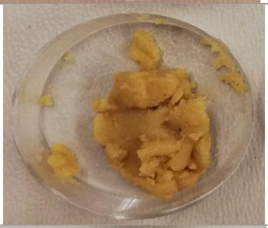
**C**	1.4 g		0.6 g (30%)	Consistency of dry pulp. After 24 h, it became very compact and sticky.	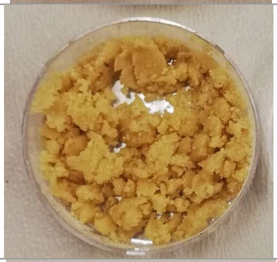
**D**	1.6 g		0.4 (20%)	Sandy and dry texture, difficult to obtain a homogeneous mix	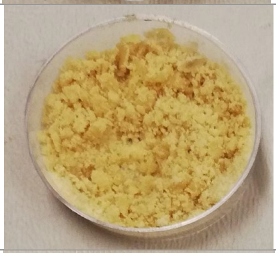
**E**	0.75 g	0.25 g	1 g	Same texture as B.	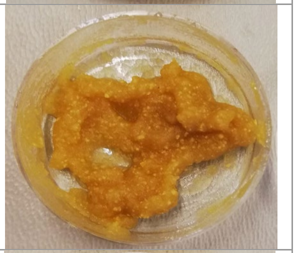
**F**	0.5 g	0.5 g	1 g	Same texture as E, with less wet consistency.	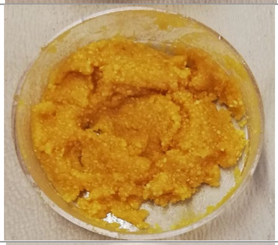
**G**	0.25 g	0.75 g	1 g	Same texture as E, with less wet consistency.	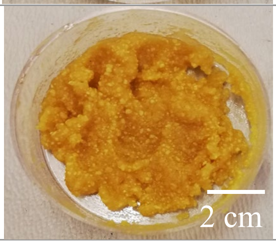

Table S2. Scoring criteria for the extruded samples according to the experimental study.

Score	Experimental observation
**0**	Not possible to extrude.
**1**	No apparent pores and discontinuous/brittle extrudate.
**2**	No apparent pores and continuous/flexible extrudate.
**3**	Few apparent pores and discontinuous/brittle extrudate.
**4**	Few apparent pores and continuous/flexible extrudate.
**5**	Presence of small and/or large pores and discontinuous/brittle extrudate.
**6**	Presence of small and/or large pores and continuous/flexible extrudate.
**7**	Large pores interconnected by smaller pores, non-homogeneous, discontinuous/brittle extrudate.
**8**	Large pores interconnected by smaller pores, non-homogeneous, continuous/flexible extrudate.
**9**	Large pores interconnected by smaller pores, homogeneous, discontinuous/brittle extrudate.
**10**	Large pores interconnected by smaller pores, non-homogeneous, continuous/flexible extrudate.

Estimate of the product price and comparison with the products available in the market

For the price estimation, we took the formulation with the most expensive components, *i.e.,* 35 % of the mixture 25 WG/75 Z; 60% glycerol (Gly) and 5% bicarbonate SB (Table 1, main article text), and based the calculation on the average prices reported on the open web for these raw materials. The price estimation included an additional 10% corresponding to costs associated with production and 10% maintenance costs. The detailed price estimation is shown in Table S3.

Table S3. Detailed cost estimation and final product price.

Reactive	Composition (%)	g/kg of product	Price USD/kg for raw materials	Price USD/kg of formulation
**Glycerol (Gly)**	60	600	4.0	2.4
**SB**	5	50	3.0	0.15
**Protein (75Z/25WG)**	35	262.5 Zein	13.0	3.41
		87.5 WG	1.7	0.15
			**Total cost**	6.11
	**Considering production and maintenance cost**			1.2*6.11
		**Total estimated product price**		**7.33**

Table S4 compares our current bio-based alternative with existing commercial superabsorbent polymers (SAP) products. For this, the following definition was considered: SAP is polymeric macromolecules that comprise a large amount of hydroxyl group, forming a three-dimensional network structure. For this hydrophilic group's presence, SAPs can absorb aqueous fluids such as water, saline solution, body fluid, etc. These types of SAP are generally based on synthetic and non-biodegradable polymers.

Table S4. Comparison of the currently developed prototype with commercial superabsorbent polymer (SAP) alternatives. Source: www.socochem.com.

**Biobased SAPs reporting in this research. Estimated price**	**SAPs (Sodium Polyacrylate) Grade: Industrial Grade (India).**	**CHEMZEST Sodium Polyacrylate**	**BFC Sodium Polyacrylate LR – 1**
**7.33 USD/Kg**	10.8 USD/Kg	7.20 USD/Kg	4.92 USD/Kg

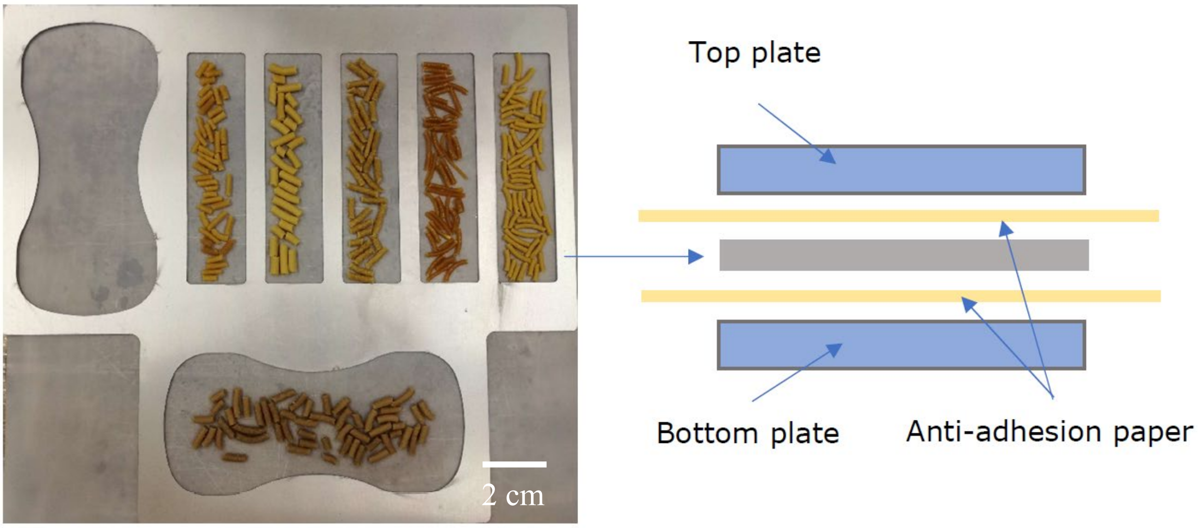

Figure S1. Hot-pressing of the porous pellets from the extruded filaments to form the different shapes towards a sanitary absorbent pad.

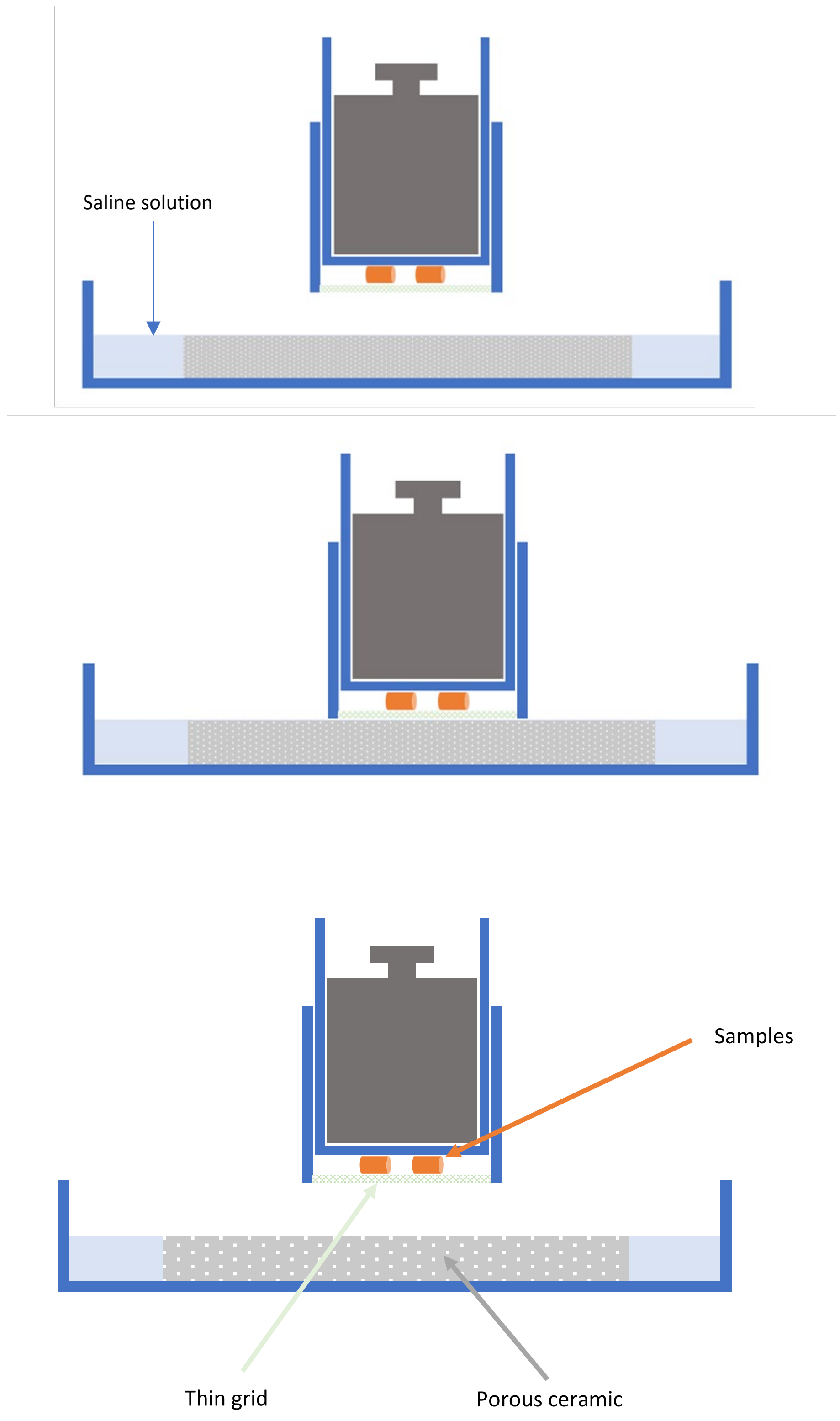

Figure S2. Absorption Under Load equipment (AUL).

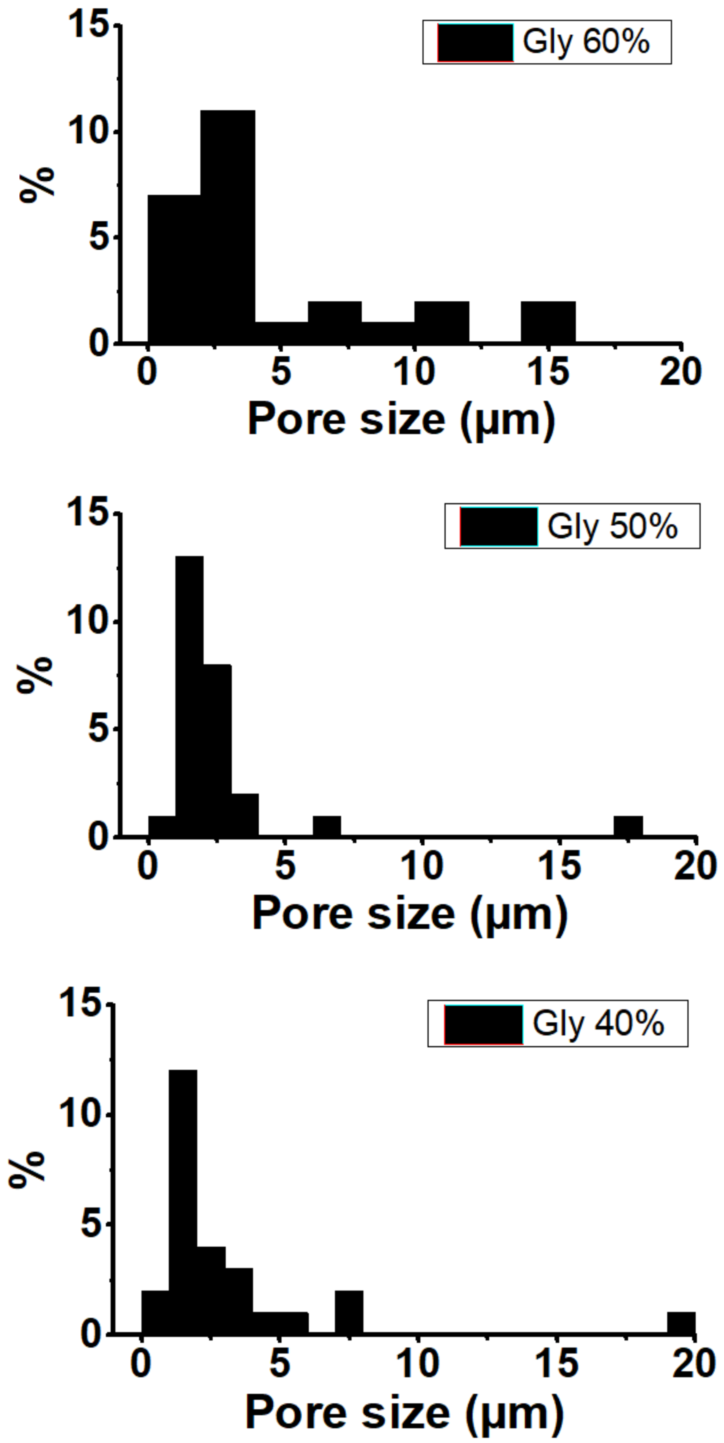

Figure S3. Pore size distribution of microporosity of the 75Z/25WG/5SB/5MQ samples (extruded at 100 ºC and 60 rpm) with different glycerol content.

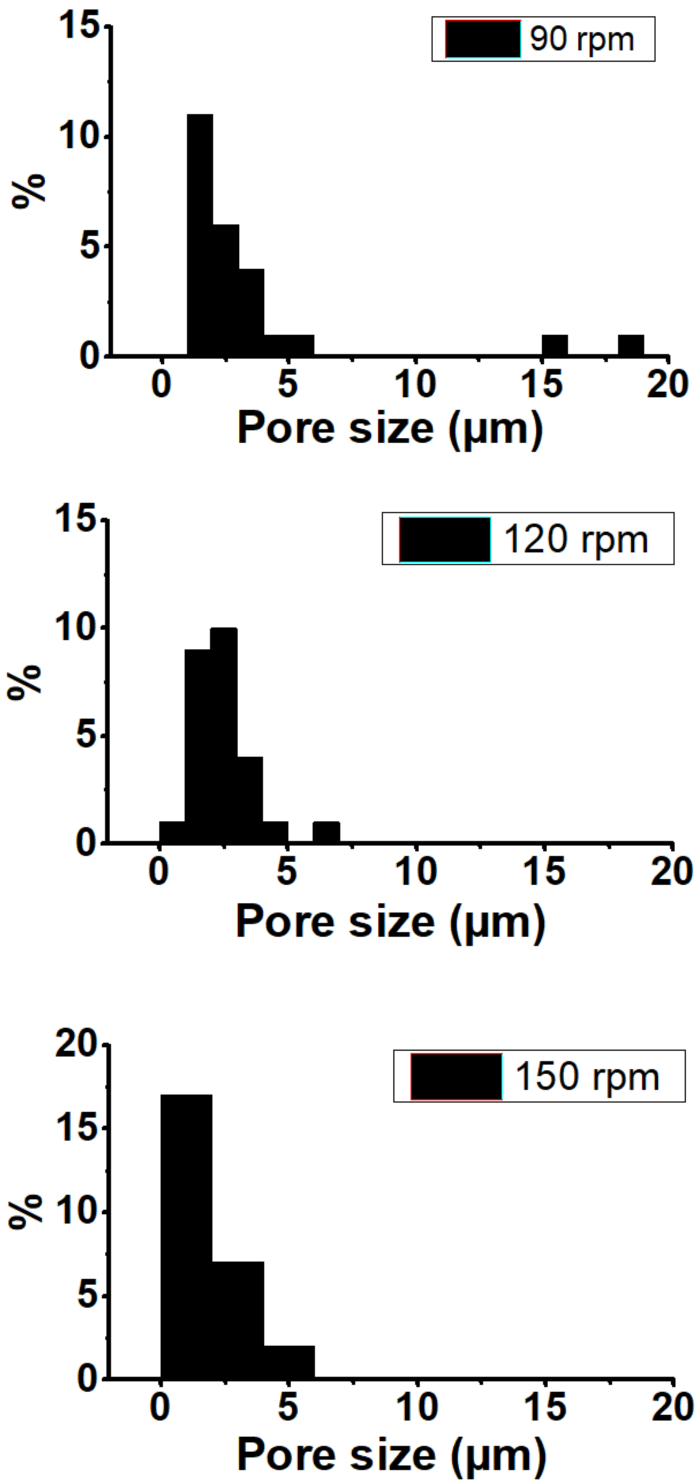

Figure S4. Pore size distribution of microporosity of the 75Z/25WG/50Gly/5SB/5MQ samples (extruded at 100 ºC) with different extrusion speeds.

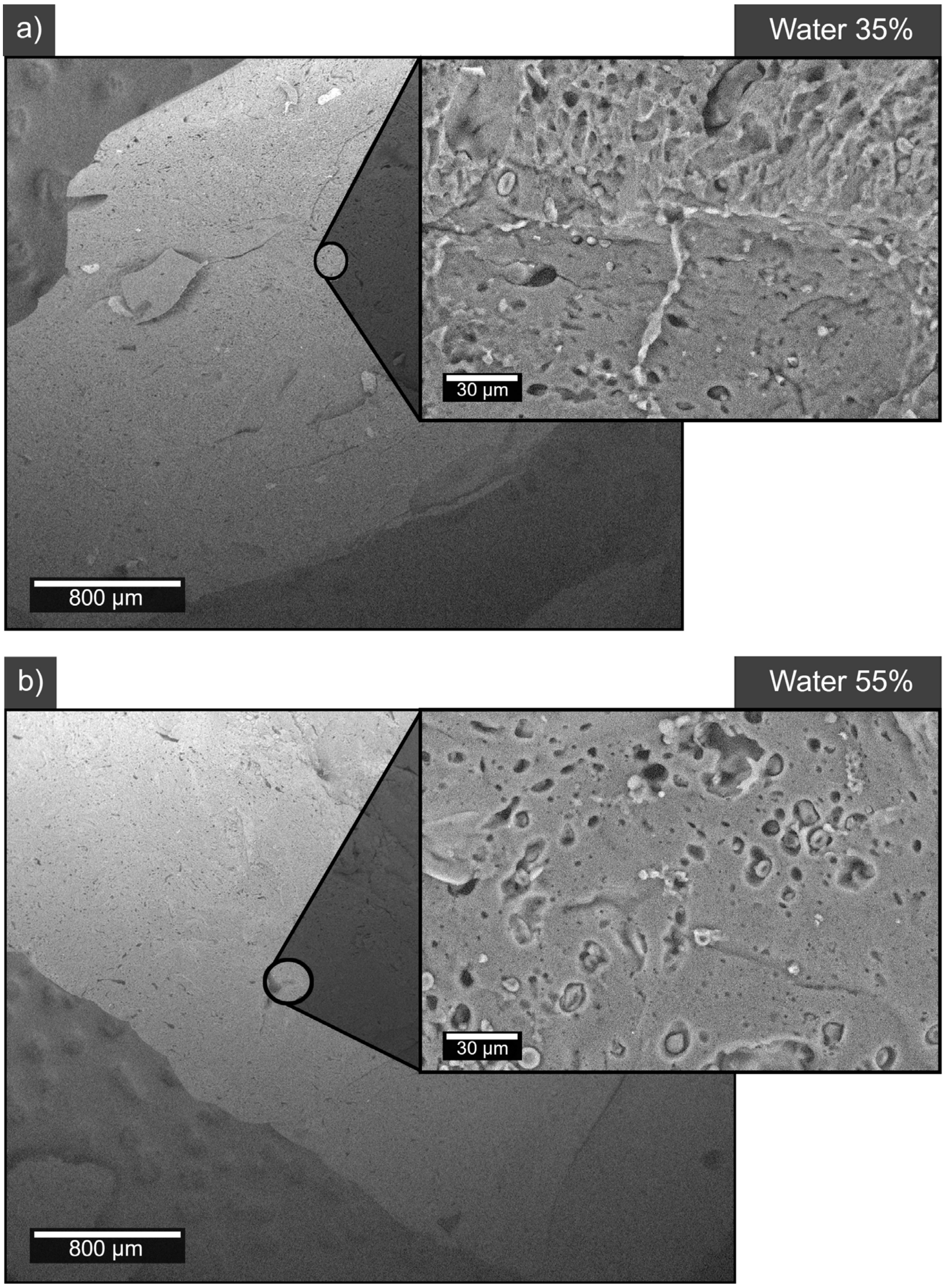

Figure S5. SEM images of the 75Z/25WG/50Gly/5SB cross-sections (extruded at 100°C) with varying water content (35 and 55 wt%, a-b, respectively).

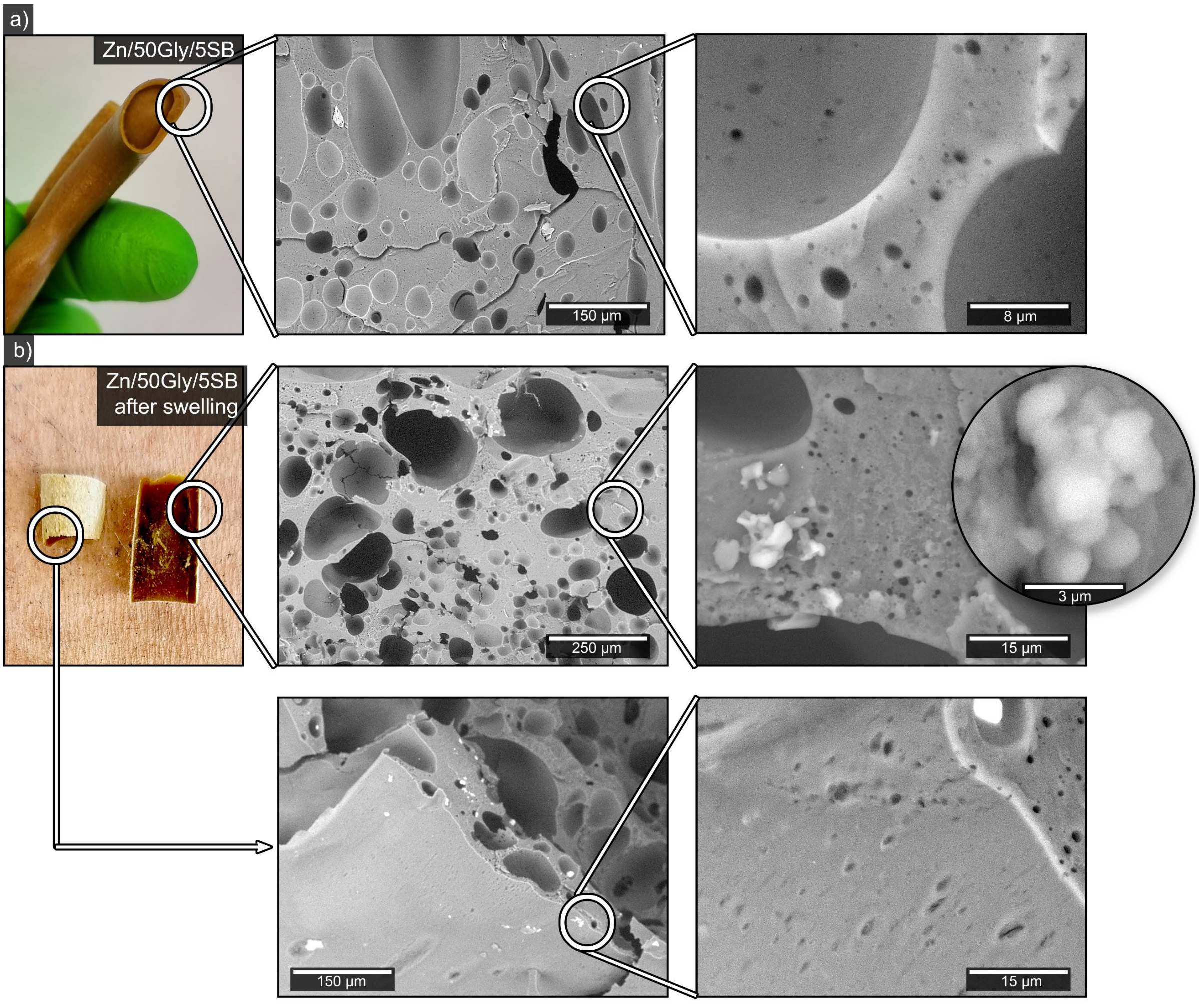

Figure S6. Pictures of the pure zein, glycerol and sodium bicarbonate extrudate (extruded at 100°C, Zn/50Gly/5SB, a). The sample was left 24 h in 0.9 wt% NaCl solution and lyophilized, and a photo of the surface and cross-section is shown in "b." The respective surface and cross-section SEM images are also shown in "b." The difference in color between the surface and the cross-section in "b" indicates a more porous structure than the inner section after the swelling, thereby indicating that the saline solution had only penetrated the outer shell of the extrudate. The inset in "b" shows agglomerates corresponding to NaCl crystals from the saline solution after swelling.

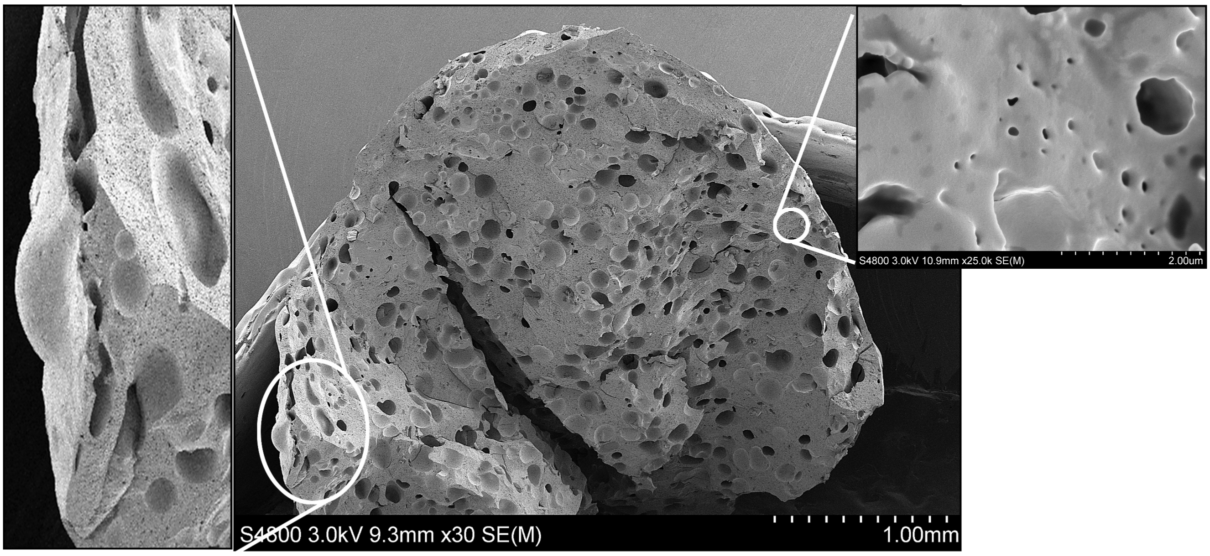

Figure S7. Cross-section SEM images of the extruded 75Z/25WG/50Gly/5SB/5MQ filament using a cylindrical die showing a solid external layer.

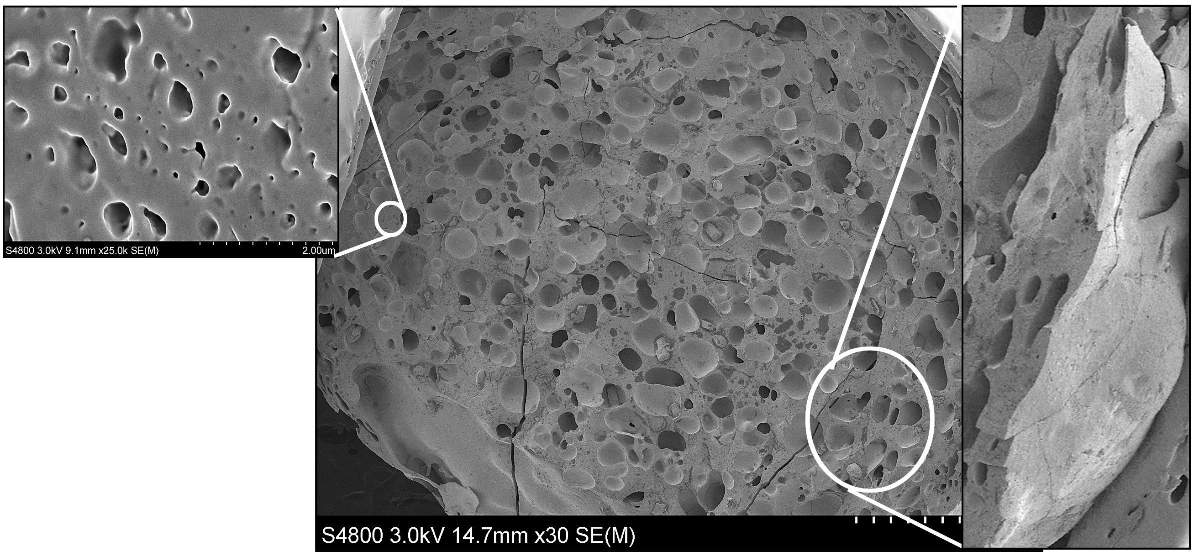

Figure S8. Cross-section SEM images of the extruded 75Z/25WG/50Gly/5SB filament using a cylindrical die showing a solid external layer.

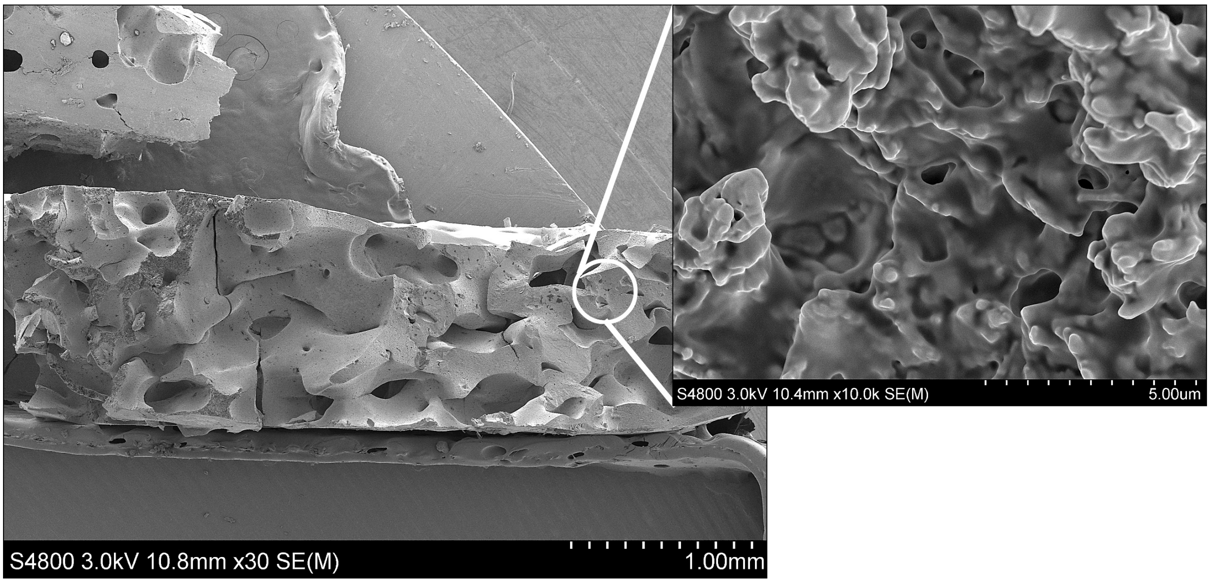

Figure S9. Cross-section SEM images of the 75Z/25WG/50Gly/5SB extruded filament chopped into pellets and hot pressed at 150 ºC (150 kN) forming a pad.

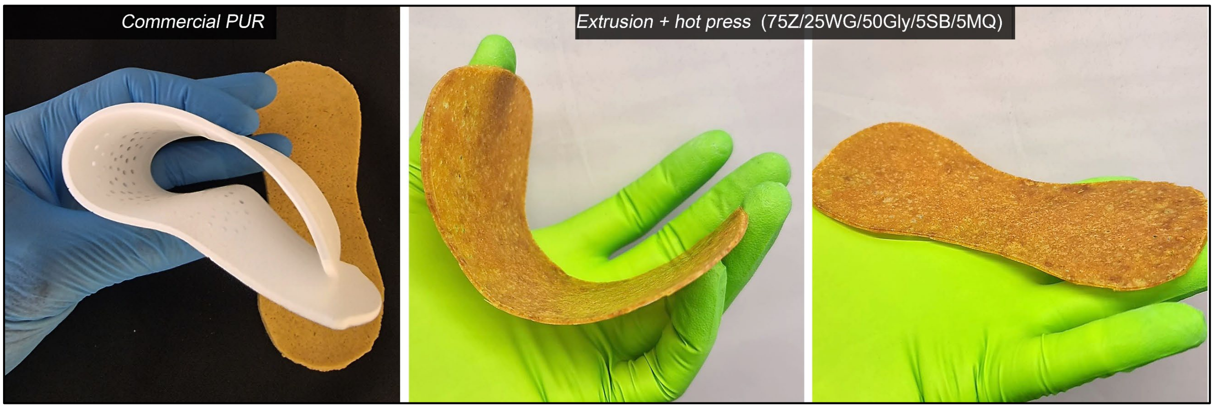

Figure S10. Commercial PUR pad removed from a commercial sanitary article and bent compared to the extrusion + hot pressed (75Z/25WG/50Gly/5SB/5MQ) pad prototype.

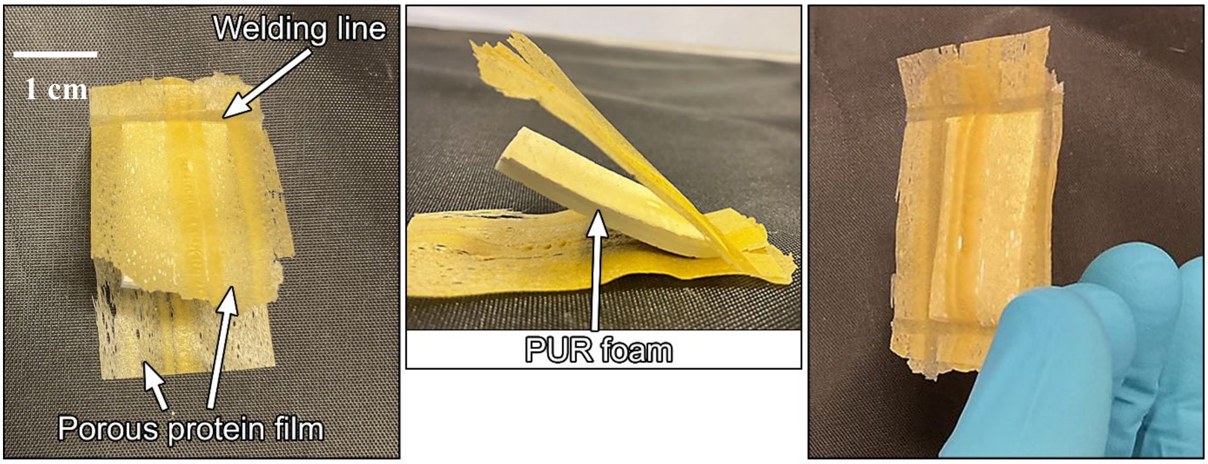

Figure S11. Picture of the 75Z/25WG/50Gly/5SB/5MQ extruded porous film between a PUR reference foam from a sanitary pad. The film was welded on one side to illustrate the sealing of the layers (left and center) and then sealed on the 4 sides (right).

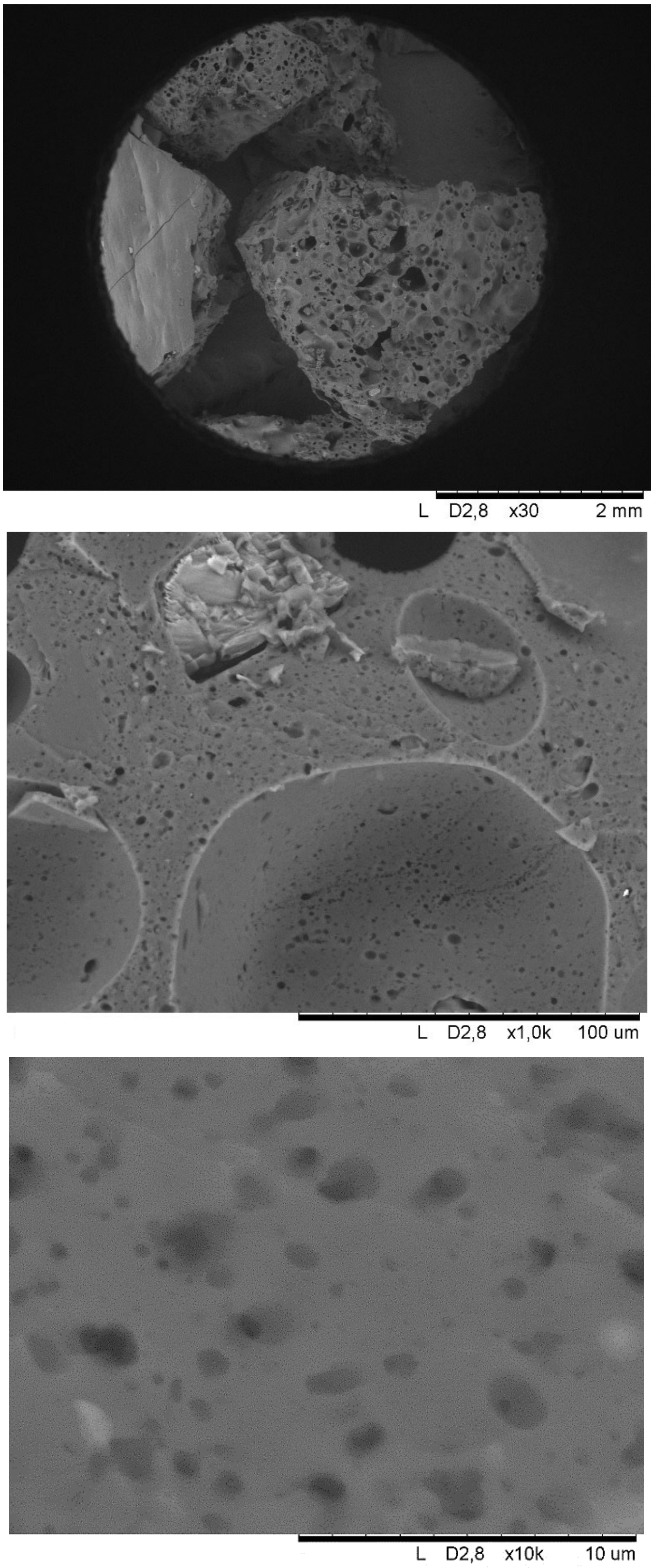

Figure S12. SEM image of the 75Z/25WG/50Gly/5SB/5MQ extrudate after grounding into porous absorbent powder showing intact microstructure.

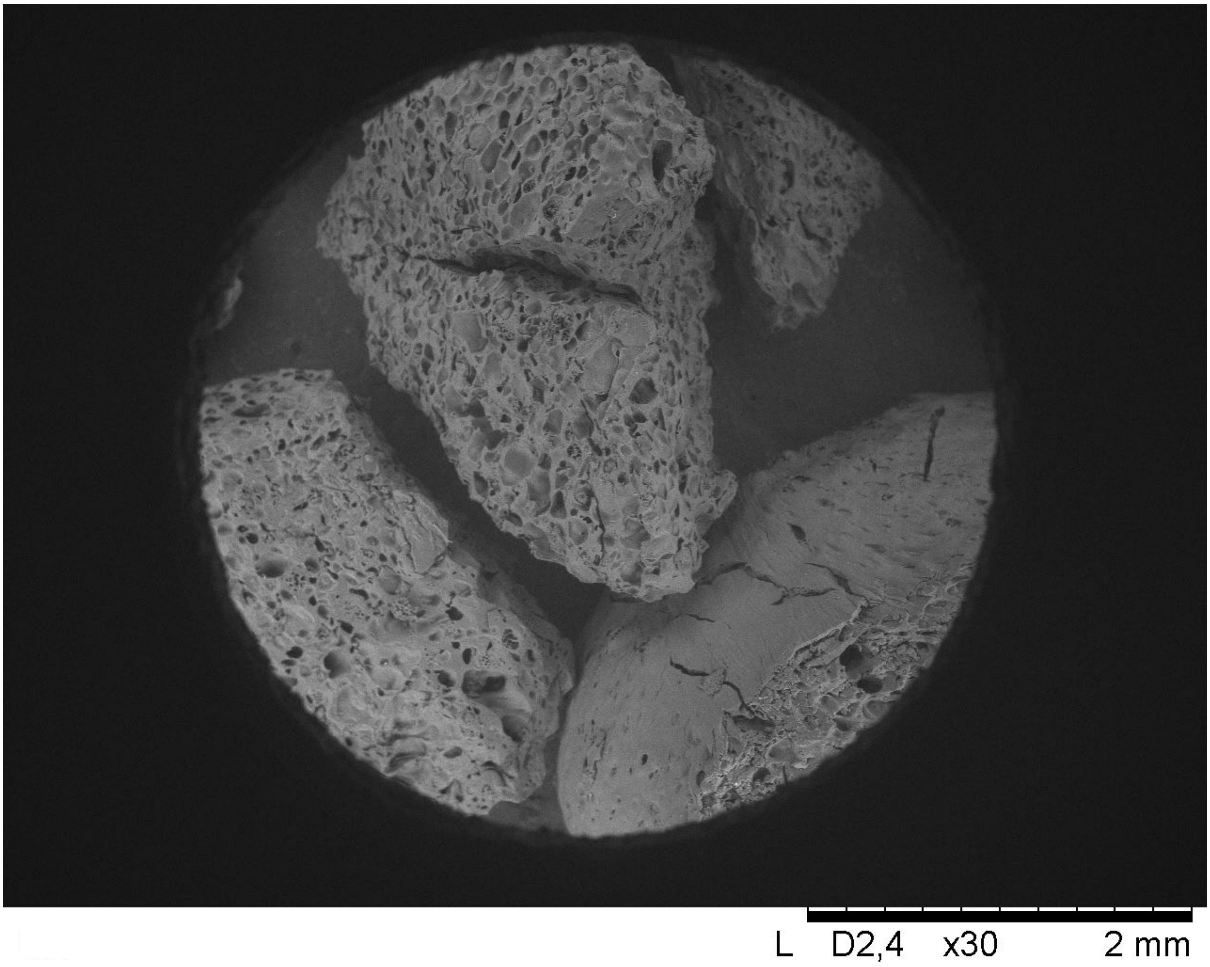

Figure S13. SEM image of the 75Z/25WG/50Gly/5SB extrudate after grounding into porous absorbent powder, exposed to 0.9% NaCl solution (FSC) for 30 min, frozen in liquid nitrogen and lyophilized.

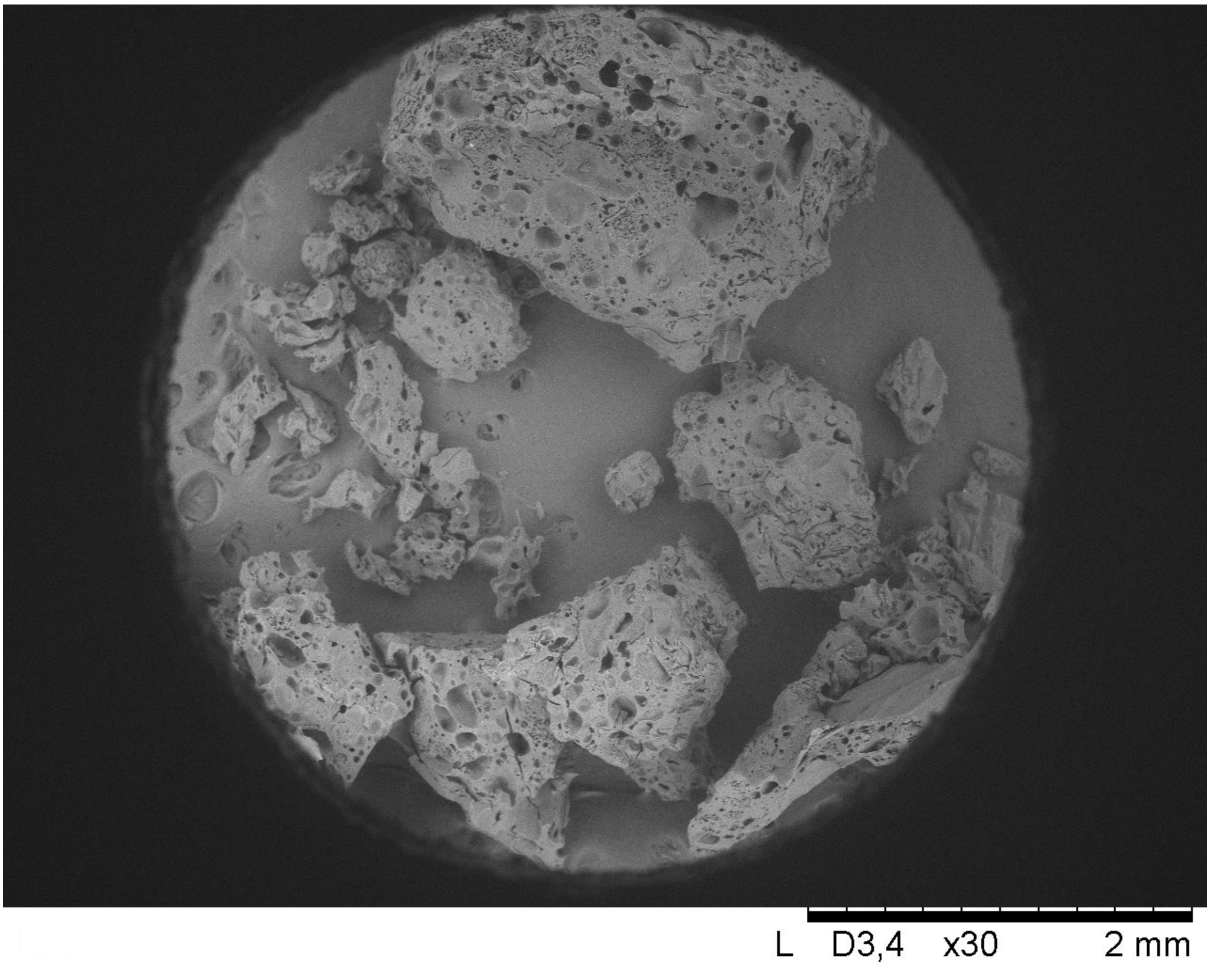

Figure S14. SEM image of the 75Z/25WG/50Gly/5SB/5MQ extrudate after grounding into porous absorbent powder, exposed to 0.9% NaCl solution (FSC) for 30 min, frozen in liquid nitrogen and lyophilized.

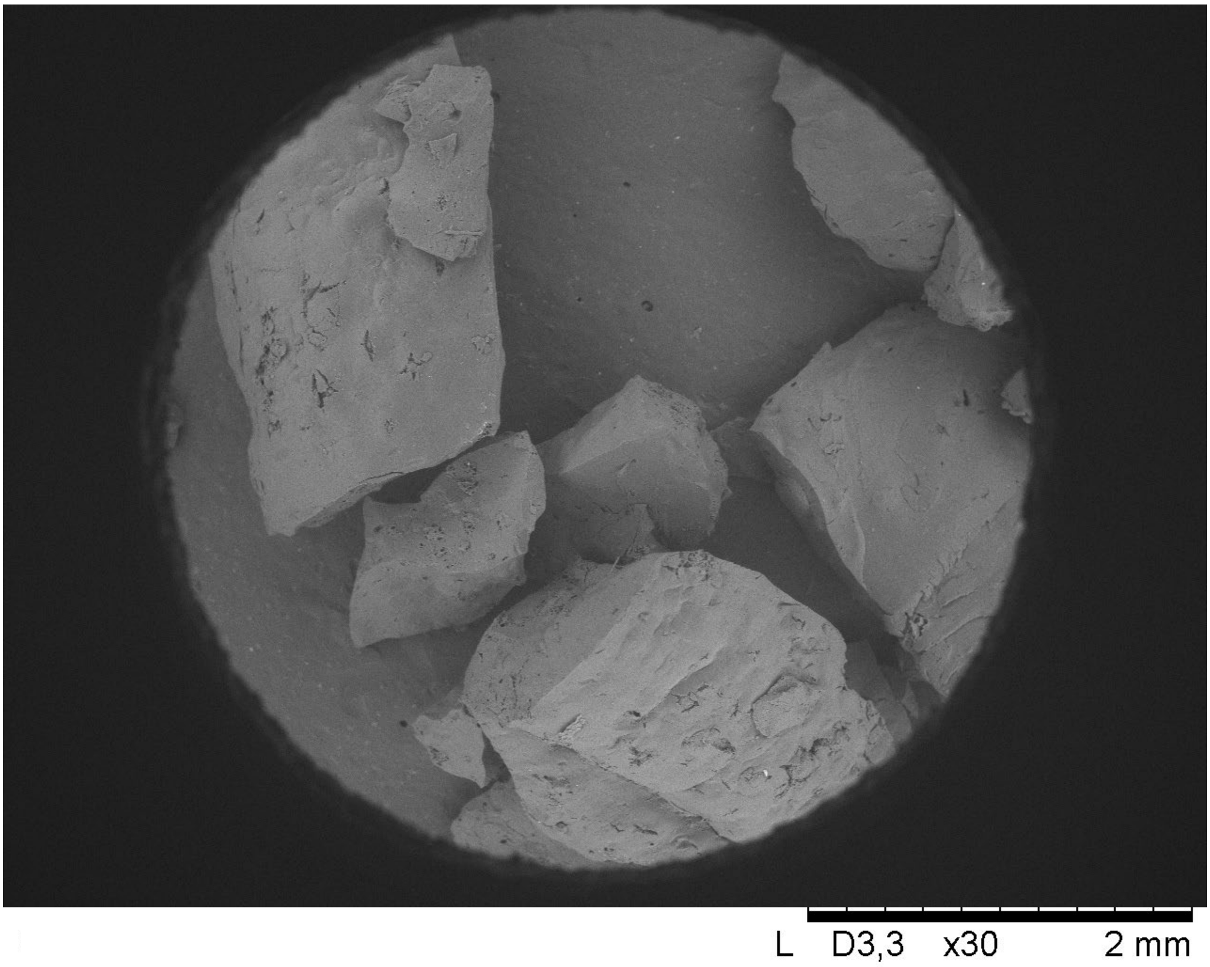

Figure S15. SEM image of the 75Z/25WG/50Gly extrudate after grounding into porous absorbent powder, exposed to 0.9% NaCl solution (FSC) for 30 min, frozen in liquid nitrogen and lyophilized.

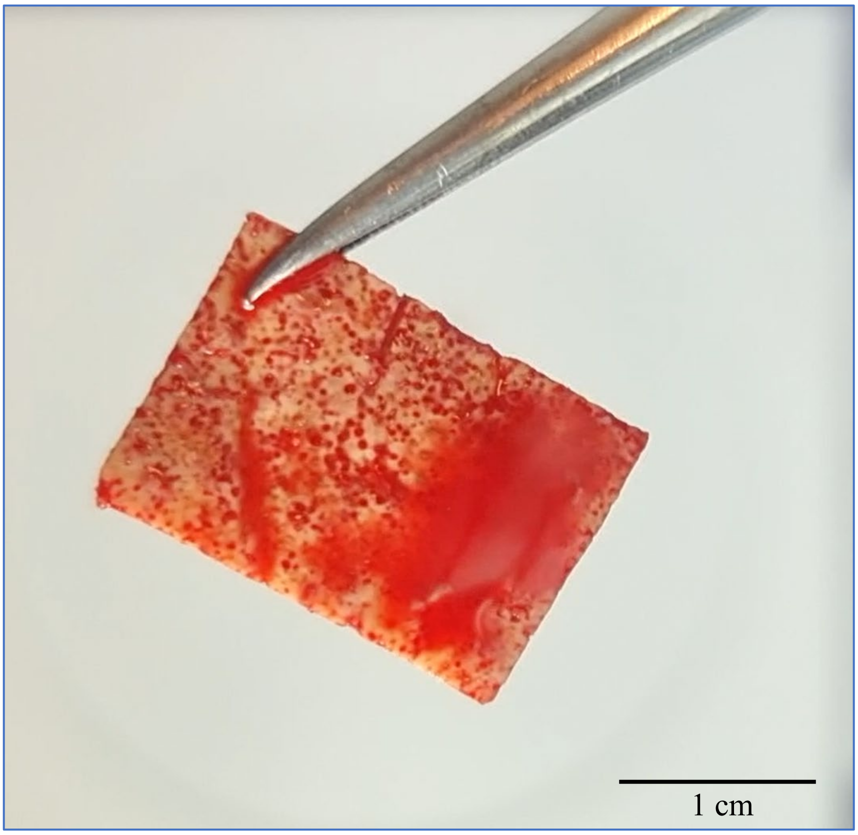

Figure S16. Compressed material (75Z/25WG/50Gly/5SB) showing the blood encapsulated in the material's pores.

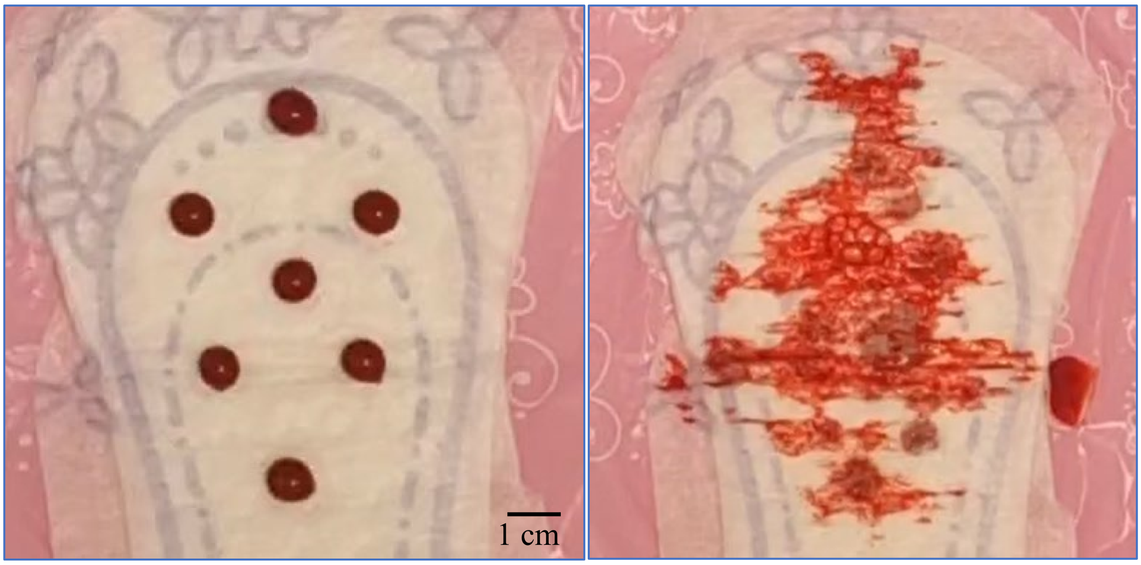

Figure S17. Images showing the defibrinated sheep blood droplets staying at the surface of the nonwoven PE layer in a commercial sanitary pad. The droplets are absorbed in the material after applying external force (left and right, respectively).

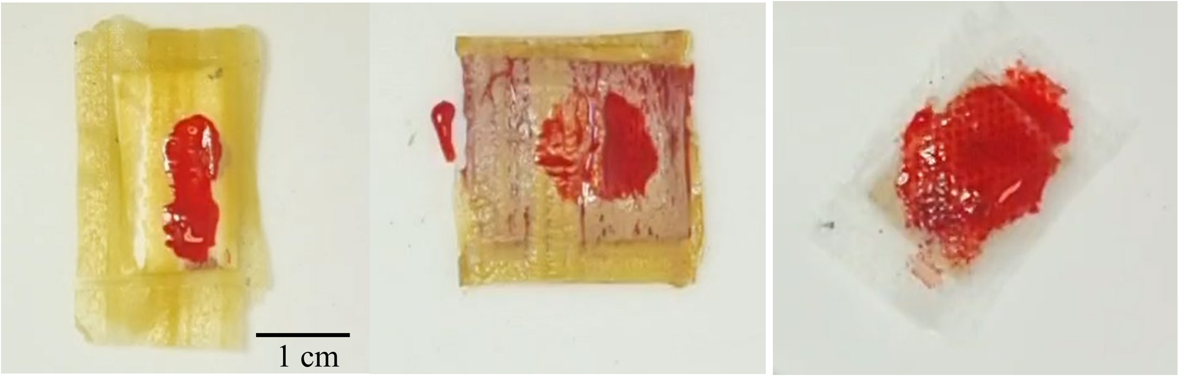

Figure S18. Images showing the different assembled materials during the VAT with defibrinated sheep blood. (Left) The porous protein film encapsulates the synthetic polyurethane foam (reference material), and the droplets are absorbed within the structure. (Center) Porous nonwoven protein film encapsulating a protein foam. (Right) Porous nonwoven (PE – reference material) encapsulating the protein foam, where external force was applied for the droplet to go through the material.

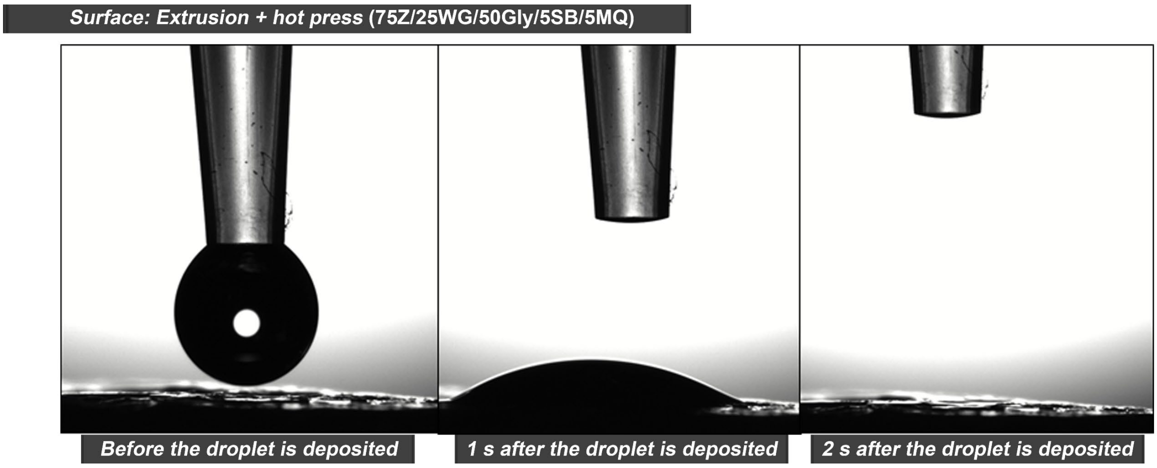

Figure S19. Contact angle screenshots of the extrusion + hot pressed sample 75Z/25WG/50Gly/5SB/5MQ showing their high hydrophilicity/wettability.

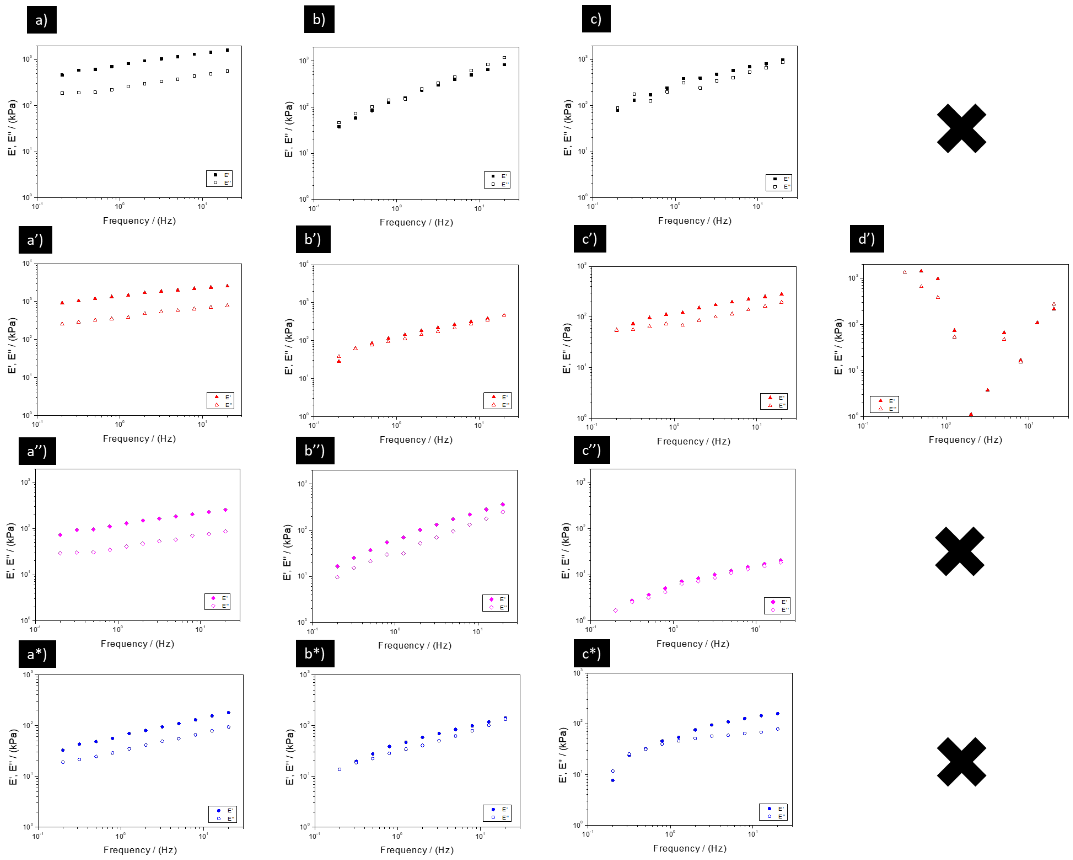

Figure S20. Frequency sweep tests of zein-gluten (black), zein-gluten-bicarbonate (red), zein-gluten-water (pink) and zein-gluten-bicarbonate-water (blue) blends at 20 ºC (A, A', A'' and A*, respectively), 80 ºC (B, B', B'' and B*, respectively), 100 ºC (C, C', C'' and C*, respectively) and 140 ºC (D').

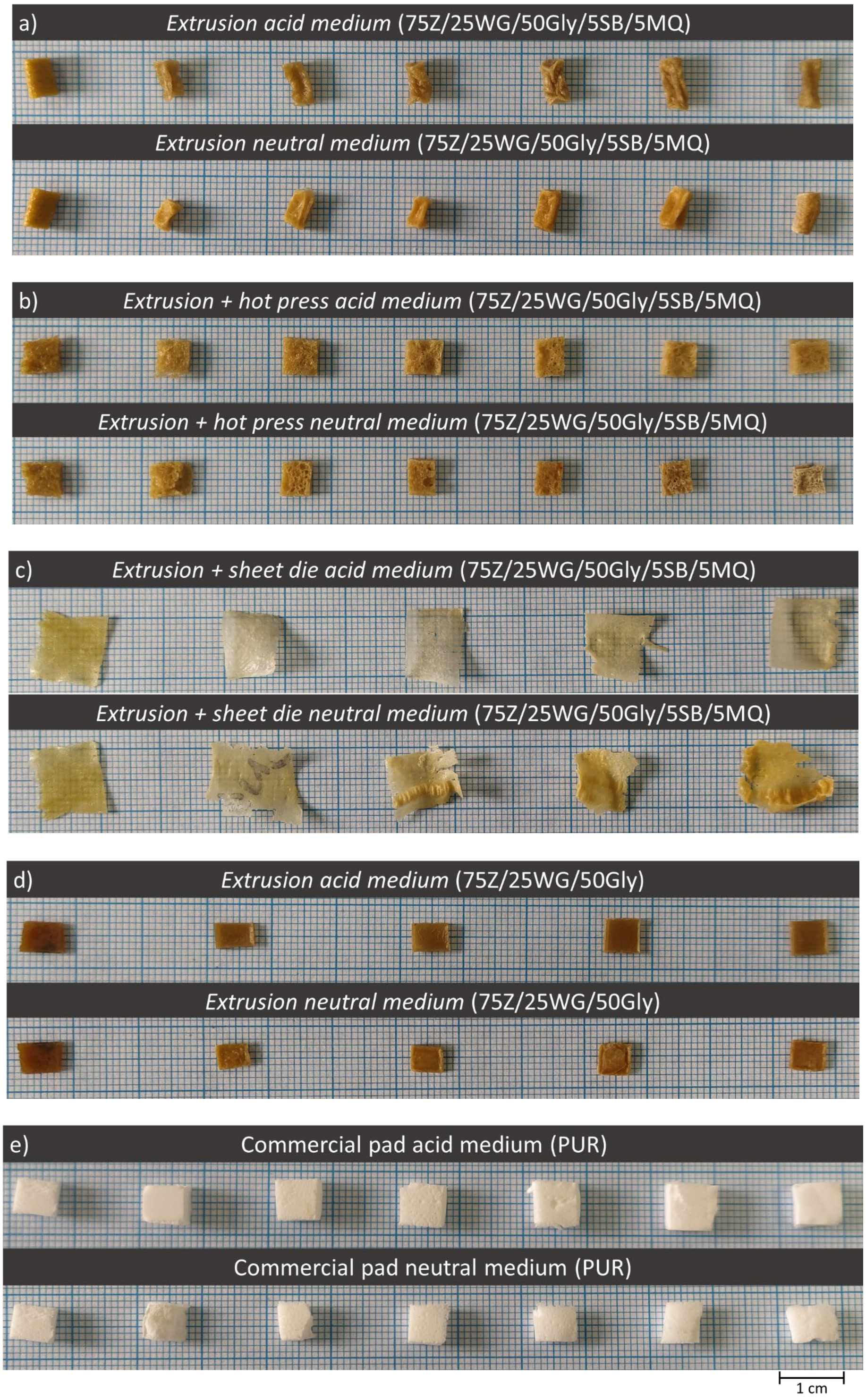

Figure S21. Images of the visual aspect of the different materials (a) Extrusion (75Z/25WG/50Gly/5SB/5MQ) (b) Extrusion + hot press (75Z/25WG/50Gly/5SB/5MQ) (c) Extrusion + sheet die (75Z/25WG/50Gly/5SB/5MQ) (d) Extrusion (75Z/25WG/50Gly) (e) Commercial pad (PUR) on the acid (top) and neutral (bottom) mediums.

a)

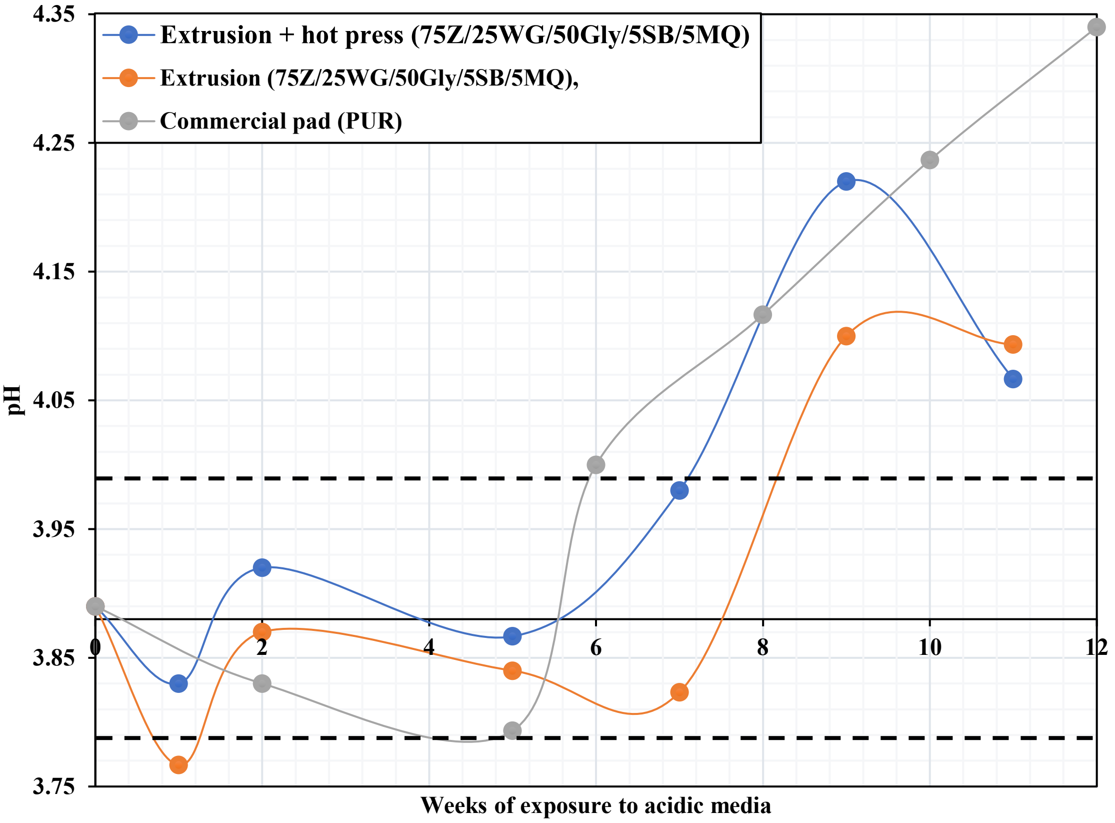

4.35
4.25
4.15
4.05
3.95
3.85
3.75
pH
0
2
4
6
8
10
12
Extrusion + hot press (75Z/25WG/50Gly/5SB/5MQ)
Extrusion (75Z/25WG/50Gly/5SB/5MQ),
Commercial pad (PUR)
Weeks of exposure to acidic media

b)

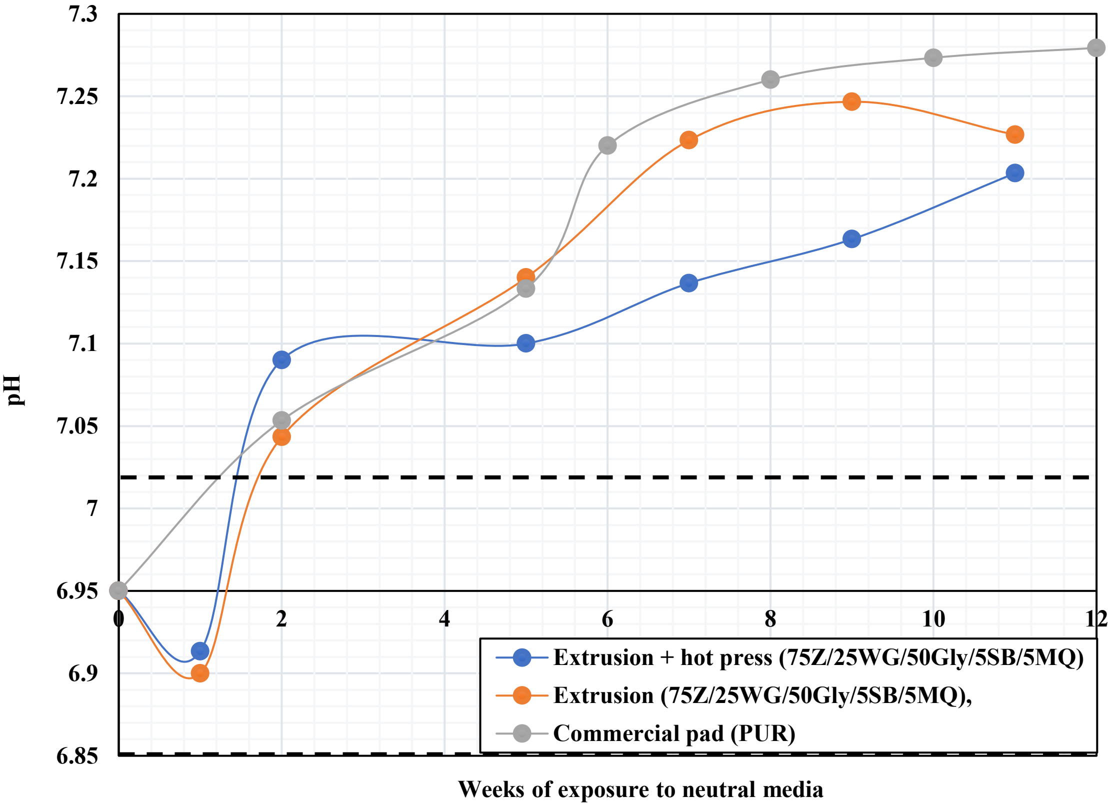

7.3
7.25
7.2
7.15
7.1
7.05
7
6.95
6.9
6.85
pH
0
2
4
6
8
10
12
Extrusion + hot press (75Z/25WG/50Gly/5SB/5MQ)
Extrusion (75Z/25WG/50Gly/5SB/5MQ),
Commercial pad (PUR)
Weeks of exposure to neutral media

c)

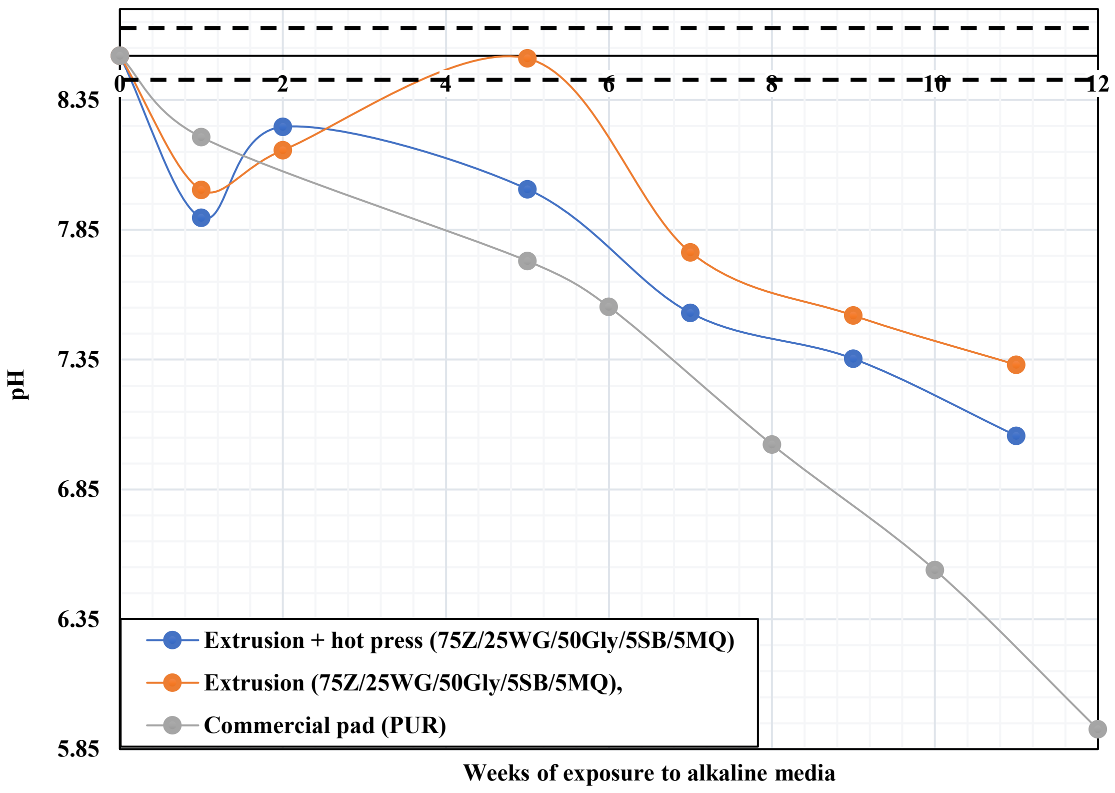

Figure S22. Plot showing the pH changes of the supernatant over the different degradation times in (a) acid, (b) neutral and (c) alkaline mediums for the Extrusion (75Z/25WG/50Gly/5SB/5MQ), Extrusion + hot press (75Z/25WG/50Gly/5SB/5MQ) and Commercial pad (PUR) (dotted lines marks the error associated to the measurements pH = ± 0.10). Due to similar values obtained for all protein-blend products, results are only shown for two of them.
